# Identification of QTNs and Their Candidate Genes for 100-Seed Weight in Soybean (*Glycine max* L.) Using Multi-Locus Genome-Wide Association Studies

**DOI:** 10.3390/genes11070714

**Published:** 2020-06-27

**Authors:** Muhammad Ikram, Xu Han, Jian-Fang Zuo, Jian Song, Chun-Yu Han, Ya-Wen Zhang, Yuan-Ming Zhang

**Affiliations:** 1 Crop Information Center, College of Plant Science and Technology, Huazhong Agricultural University, Wuhan 430070, China; ikramuaf35@gmail.com (M.I.); xuhan@webmail.hzau.edu.cn (X.H.); jfzuo@webmail.hzau.edu.cn (J.-F.Z.); hanchunyu@webmail.hzau.edu.com (C.-Y.H.); yawen@webmail.hzau.edu.cn (Y.-W.Z.); 2 College of Agriculture, Nanjing Agricultural University, Nanjing 210095, China; ahszsongjian@163.com

**Keywords:** 100-seed weight, marker assisted selection, multi-locus genome-wide association studies, quantitative trait nucleotide, single nucleotide polymorphisms, soybean

## Abstract

100-seed weight (100-SW) in soybeans is a yield component trait and controlled by multiple genes with different effects, but limited information is available for its quantitative trait nucleotides (QTNs) and candidate genes. To better understand the genetic architecture underlying the trait and improve the precision of marker-assisted selection, a total of 43,834 single nucleotide polymorphisms (SNPs) in 250 soybean accessions were used to identify significant QTNs for 100-SW in four environments and their BLUP values using six multi-locus and one single-locus genome-wide association study methods. As a result, a total of 218 significant QTNs were detected using multi-locus methods, whereas eight QTNs were identified by a single-locus method. Among 43 QTNs or QTN clusters identified repeatedly across various environments and/or approaches, all of them exhibited significant trait differences between their corresponding alleles, 33 were found in the genomic region of previously reported QTLs, 10 were identified as new QTNs, and three (*qHSW-4-1*, *qcHSW-7-3*, and *qcHSW-10-4*) were detected in all the four environments. The number of seed weight (SW) increasing alleles for each accession ranged from 8 (18.6%) to 36 (83.72%), and three accessions (Yixingwuhuangdou, Nannong 95C-5, and Yafanzaodou) had more than 35 SW increasing alleles. Among 36 homologous seed-weight genes in *Arabidopsis* underlying the above 43 stable QTNs, more importantly, *Glyma05g34120*, *GmCRY1*, and *GmCPK11* had known seed-size/weight-related genes in soybean, and *Glyma07g07850*, *Glyma10g03440*, and *Glyma10g36070* were candidate genes identified in this study. These results provide useful information for genetic foundation, marker-assisted selection, genomic prediction, and functional genomics of 100-SW.

## 1. Introduction

Soybean (*Glycine max* L. Merr.), which provides 69% dietary protein and 30% oil [1], is economically imperative food and oilseed crop worldwide. The 100 seed weight (100-SW) is an essential trait in soybean yield component, affected by seed size and shape, and positively correlates with seed yield [2]. There are numerous soybean food items for various seed sizes, for example, large seeds are used for tofu and miso, while small seeds are used for natto [3]. After domestication, cultivated soybean exhibit diverse traits from wild soybean, and 100-SW for *G. max* is almost 6–7 fold greater than *Glycine soja* [4,5,6]. Seed weight (SW) is a quantitatively inherited trait and controlled by multiple genes, with various main and epistatic effects [2,7,8,9], and significantly influenced by the growing environmental conditions. Due to the quantitative nature, it is difficult to develop superior cultivars by traditional breeding. Thus, understanding the genetic basis of 100-SW and incorporating new breeding technologies would be helpful in the development of superior cultivars that can be used for industry and market requirements, as well as world food requirements.

The traditional selection methods in crop breeding have some drawbacks, such as being labor-intensive, high-cost, and time consuming, and they require multiple environments for several years to evaluate the materials [10]. Molecular markers are a powerful tool for soybean breeders to find the new source of genetic variation and to dissect the genetic bases of desired traits [11]. Marker-assisted selection (MAS) has clear advantages over traditional breeding by reducing the number of lines to be tested in a selection [12]. Therefore, it is important to identify quantitative trait nucleotides/loci (QTNs/QTLs) controlling seed weight to develop the superior cultivars. To date, more than 200 QTLs scattered on 20 chromosomes have been reported for SW in soybean database (www.soybase.org). These loci were identified from more than 40 different genetic populations and 50 bi-parental materials using SSR and RFLP markers [13,14]. Likewise, Xie et al. [15] identified seven SW QTLs using 22 simple sequence repeat (SSR) and 160 single nucleotide polymorphism (SNP) markers in 504 recombinant inbred lines (RILs, F_7:8_) from the direct and reciprocal crosses of Lishuizhongzihuang with Nannong493-1, and 265 soybean accessions, respectively. Similarly, Mansur et al. [16] detected QTLs for SW using restriction fragment length polymorphism markers in the genetic population from the cross of Minsoy 9 and Noir1, while Teng et al. [17] identified 42 SW QTLs. However, the identified QTLs have a large genomic region, which has relatively low accuracy due to limited recombination and low marker density. Therefore, previously reported SW-associated QTLs were not enough to identify the candidate genes [18], and also had limited applications and hindered breeding efforts to improve SW in soybean through MAS.

Recently, sequencing costs of SNPs have been drastically reduced due to the development of sequencing technologies [19]. Thus, genome-wide association studies (GWAS) can be used for QTN detection in natural populations with high-density markers to overcome the limitation of bi-parental QTL mapping [20]. In other words, a QTN is a nucleotide polymorphism that is predicted to be responsible for the observed variation of complex trait [21]. GWAS has multiple advantages over linkage analysis, such as high power, more alleles, and large variation [22,23]. Up to now, there have been several GWAS articles in the detection of SW QTNs in soybeans. For instance, 19 QTNs were identified for yield components in soybean landraces [24], eight domesticated QTNs were found to be associated with seed size traits [25], and 22 QTNs and their candidate genes were found to be associated with seed weight [26]. Recently, Yan et al. [27], Jing et al. [28], Zhao et al. [8], Li et al. [7], Assefa et al. [29], and Hu et al. [30] identified 17, 33, 34, 63, 14, and 34 QTNs, respectively, for 100-SW in soybean.

In *Arabidopsis*, some genes such as *MINI3, SHB1, IKU1, IKU2, AP2, OBP1*, and *AFR2* have been functionally characterized for seed development and size [31,32,33,34,35]. In soybean, two genes *GmCYP78A5* and *GmGA20OX* were cloned, and these genes increased seed size/weight in transgenic plants [36,37]. Likewise, Lu et al. [38] identified soybean gene *Glyma17g33690*, which encodes the phosphatase 2C protein-1 (PP2C-1). The PP2C-1 increased 100-SW in transgenic plants. Furthermore, Wang et al. [39] determined three candidate genes (*Glyma18g05240*, *Glyma11g05760*, and *Glyma18g43500*) for seed weight, based on homologous genes in *Arabidopsis* and rice. Similarly, Gu et al. [40] identified *SoyWRKY15a* as a candidate gene for seed size, and its orthologous genes *GmWRKY15a* (*G. max*) and *GsWRKY15a* (*G. soja*) were associated with seed weight. Five candidate genes, *GmRGI-3* [41], *Glyma06g43880* [42], *Glyma02g41270* [43], *Glyma05g34120* [7], and *Glyma15g05650* [44] have been identified to be associated with seed development, lower seed yield, 100-SW, small seed, and seed development in soybean, respectively. Recently, gene *GsCID1* (*Glysoja.04g010563*) was identified to be associated with SW and highly expressed during seed developmental stages [30].

To date, several studies reported QTLs/QTNs regarding soybean SW using linkage and association mapping, but the related genes in soybean are relatively limited. The possible reason for this, firstly, a high degree of linkage disequilibrium (LD) in the soybean genome makes it difficult to detect the QTNs and genes precisely. Secondly, the number of molecular markers used in soybean GWAS is relatively small with low density, which reduces the efficiency of GWAS. Thirdly, single-locus GWAS (SL-GWAS) models were used in soybean for yield traits [45,46,47], and these models are single-locus genome-wide scanning and need multiple tests correction (e.g., Bonferroni correction) that removes many significant small-effect QTNs [23]. To overcome these limitations, Zhang’s group developed a series of multi-locus GWAS (ML-GWAS) methods, such as mrMLM [22], and these ML-GWAS methods were used to dissect the genetic foundations of complex traits in different crops [23,48,49,50]. Currently, most of the studies have used SL-GWAS methods for the detection of 100-SW QTNs. However, almost no ML-GWAS articles have been found to detect QTNs for 100-SW in soybean. Therefore, more efficient studies are required to dissect the genetic basis of 100-SW, and exploring the QTNs/candidate genes associated with SW will be paramount for the genetic improvement and production of this crop.

Therefore, the objectives of this study were: (a) to dissect the genetic basis for 100-SW using the ML-GWAS methods, and compare the QTN results with those in all the previous studies; (b) to identify the seed weight (SW) increasing alleles of these QTNs for MAS, and (c) to find the potential candidate genes regulating 100-SW in the region of stable QTNs. The findings in this study will provide reliable information for MAS in soybean breeding and functional gene validation/cloning.

## 2. Materials and Methods

### 2.1. Plant Materials

A total of 250 soybean accessions were selected from different geographic regions of China. These soybean accessions came from 23 provinces and were disseminated in six eco-regions of China [25], and were obtained from the National Center for Soybean Improvement and Linyi Academy of Agricultural Sciences with 139 landraces and 111 cultivars.

All the accessions were planted at the Jiangpu Experimental Station of Nanjing Agricultural University (from June to October) and Experimental Station of Huazhong Agricultural University (from May to October) in 2014 (denoted as E1 and E3) and 2015 (E2 and E4). Plants were grown in 150 cm wide and 200 cm long plots according to the randomized complete block design with three replicates. The flowering time was started after six to eight weeks of emergence. The trait phenotypes were measured from five plants in the middle row of each plot, and 100-SW for each accession was averaged based on three replicates.

### 2.2. Statistical Analysis and Heritability Estimation

The best linear unbiased prediction (BLUP) of 100-SW for each accession was calculated using the R (http://www.R-project.org/, v3.5.0) package lme4 [51] with the following model:

Phenotype~(1|Genotype) + (1|Year)

The aov function in the R software was used to calculate the variances of 100-SW, and mixed linear model (MLM) was used to estimate polygenic variance components and heritability [22] with the following equation:

y=Xα+ϕ+ε

where *y* is the phenotypic vector; *X* is an incident matrix for fixed (non-genetic) effects, and α is a vector of fixed effects; $\phi \sim \mathrm{MVN}(0,K\sigma_{g}^{2})$ is the polygenic effect with a multivariate normal distribution with zero mean, $\sigma_{g}^{2}$ is polygenic variance, and kinship matrix **K** was calculated from marker information [52]; $\varepsilon \sim \mathrm{MVN}(0,I\sigma_{e}^{2})$ is the vector of residues, and $\sigma_{e}^{2}$ was residual variance. The above two variance components were estimated from restricted maximum likelihood [53]. The broad-sense heritability was calculated using the following equation:

$$h_{B}^{2}=\frac{\sigma_{g}^{2}}{\sigma_{g}^{2}+\sigma_{e}^{2}}$$

### 2.3. Population Structure Analysis and Genome-Wide Association Studies

RAD-seq was used to obtain high-density SNPs, while RAD-seq, genotyping of soybean accessions, methods of sequencing data calling variations, and the quality control were described in Zhou et al. [25]. In this study, a total of 43,834 SNPs with minor allele frequency (MAF) > 0.05 were used to construct a population structure using the STRUCTURE 2.3.4 software [54]. The hypothetical number of subgroups (*k*) ranged from 1 to 10. The length of the burn-in period for each run was set to 10,000, and the number of Markov chain Monte Carlo replications after burn-in was set to 100,000. The best *k* in this population was identified according to Evanno et al. [55] using STRUCTURE HARVESTER [56]. Six ML-GWAS approaches with population structure (Q) and kinship (K) were used to detect significant QTNs, including mrMLM [22], FASTmrEMMA [20], pLARmEB [57], ISIS EM-BLASSO [58], FASTmrMLM [59], and pKWmEB [60]. These methods were included in package mrMLM (https://cran.r-project.org/web/packages/mrMLM/index.html, v4.0). In the above methods, the first step was to select all the potentially associated markers, and kinship matrix K was automatically calculated. In the second step, the effects of all the selected markers were estimated by empirical Bayesian, the significances of the effects apart from zero were obtained by likelihood ratio test, and the threshold level LOD ≥ 3 (*p* = 0.0002) was used to determine significant QTNs [20,22,23,50,57,58,59,60].

### 2.4. Elite Allele Analysis

Based on the QTN effect value and code 1 for genotype, SW increasing alleles of each stable QTN can be determined. If the QTN effect value is positive, the genotype with the code of 1 is regarded as the SW increasing allele; if the QTN effect value is negative, then alternative genotype is viewed as the SW increasing allele [22,23]. The average seed weight of the accessions with one allele was calculated to verify the QTN [61]. For each QTN, the SW increasing allele percentage in mapping population was measured as the number of accessions having SW increasing allele divided by the total number of accessions. The SW increasing allele percentage for each accession was equal to the number of SW increasing alleles divided by the total number of stable QTNs. Using the stable QTN information, the best cross combinations were predicted for the soybean breeding program. If we want to add seed weight, SW increasing allele is elite allele, while SW decreasing allele is elite allele if we want to decrease seed weight.

### 2.5. Prediction of Candidate Genes for 100-Seed Weight

Prediction of candidate genes for 100-SW was performed in 100 kb downstream and upstream of each stable QTN in SoyBase (http://soybase.org/; Wm82.a1.v1.1). For the screening of genes, the transcriptomic datasets of seven different seed developmental stages such as 4, 12–14, 22–24 (DAF: Days after flowering), seed weight 5–6 mg period (5–6 mgWS), cotyledon weight 100–200 mg period (100–200 mgCOT), cotyledon weight 400–500 mg period (400–500 mgCOT), and full seed maturity period (Dry seed) of soybean Williams 82 [62] were retrieved from the Gene Expression Omnibus database (https://www.ncbi.nlm.nih.gov/geo/; accession no GSE42871). This is because genes with high RPKM at these stages are related to seed size, seed weight, cotyledon, seed coat tissues, embryo, endosperm, seed storage proteins, and seed maturation protein [63]. Thus, candidate genes were determined as below [23]. Firstly, we removed all the genes with expression level <1 in all the seven stages and selected those genes with a higher expression levels double their average expression levels in at least one seed developmental stage. Then, homologous genes related to seed weight in *Arabidopsis* were identified using BLAST analysis with the critical E value 1E-30. Finally, all homologous genes from soybean accompanied seed weight were selected, and considered candidate genes for 100-SW.

#### 2.5.1. Gene Expression Level Analysis

The freely available RNA-Seq datasets of 14 soybean tissues [63], including whole seeds from 11 stages of reproductive tissue development (flower, pod, and seed) and three vegetative tissues (leaves, root, and nodules) were obtained from SoyBase (http://soybase.org/), in order to analyze candidate genes with special higher gene expression levels in soybean seeds. The heat maps were generated by using R software packages “pheatmap”.

#### 2.5.2. Kyoto Encyclopedia of Genes and Genomes (KEGG) Pathway Analysis

The Kyoto Encyclopedia of Gene and Genomes (KEGG) enrichment analysis was conducted for potential candidate genes to identify the functional categories, implemented by KEGG Orthology-Based Annotation System network software (KOBAS v3.0) [64] (http://kobas.cbi.pku.edu.cn/kobas3), with adjusted *p* value < 0.05 as threshold criteria.

## 3. Results

### 3.1. Phenotype Variation of 100-Seed Weight

**Figure 1:**
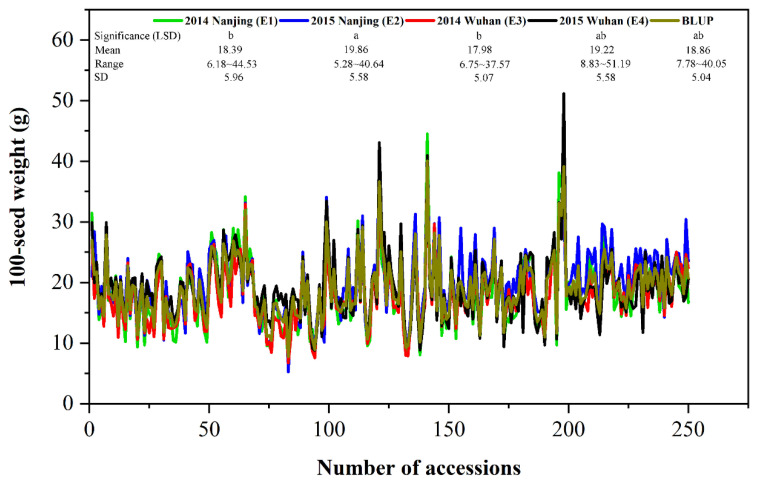
Phenotypic distributions of 100-seed weight in 250 soybean accessions in four environments and BLUP. SD: standard deviation. The significant differences of 100-seed weight among four environments/BLUP are tested by the LSD method at the 0.05 level of significance.

The 100-SW phenotype of each accession was the average of three replicates in each environment. The mean phenotypic values of 100-SW across 250 accessions in E1 to E4 environments were 18.39, 19.86, 17.98, and 19.22 (g), with standard deviations of 5.96, 5.58, 5.07, and 5.58 (g), respectively, and their coefficient of variations ranged from 28.08–29.07 (%) (Table 1). The highest phenotypic value was observed in E4, whereas the lowest phenotypic value seen in E2 (Figure 1). The continuous distribution was found in these environments (Figure 1). Two-way ANOVA showed the significant difference of 100-SW across all the accessions (*p-value* < 0.01), indicating the existence of genetic variation among these accessions (Table 1). Meanwhile, the estimates of broad-sense heritabilities (h^2^_B_) for 100-SW in E1 to E4 environments were 93.70, 88.51, 90.15, and 83.20 (%), respectively, using polygenic and residual variances (Table 1), suggesting that the genetic effects play an essential role in phenotypic variation.

### 3.2. Population Structure Analysis and Genome-Wide Association Studies

**Figure 2:**
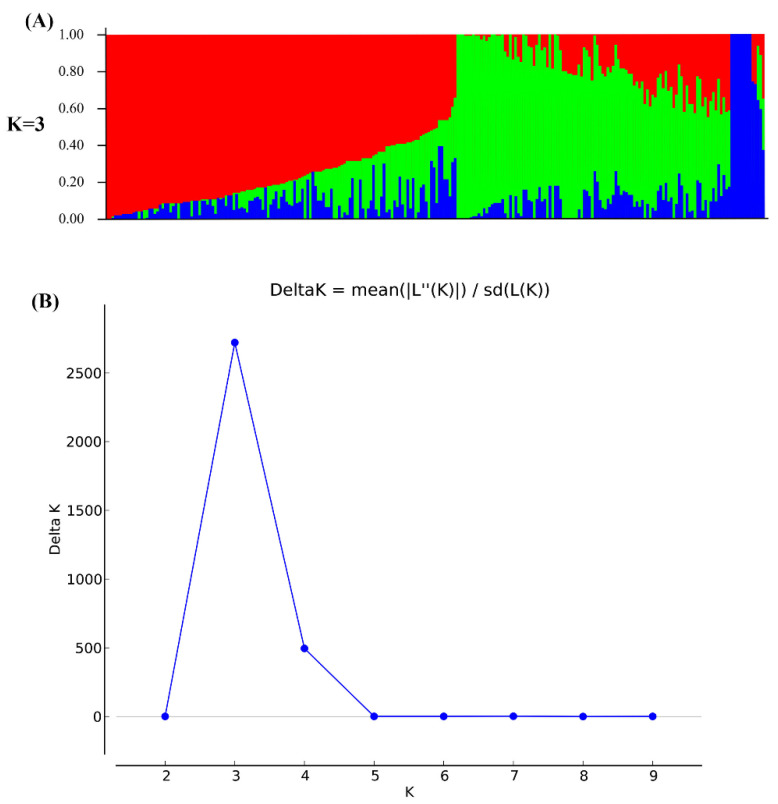
Population structure of 250 soybean accessions using 16,174 SNPs on soybean genome. (**A**) population structure (*k* = 3). Each vertical column represents one individual, and the red, green, and blue color segments in each column represents the percentages of cultivated, landrace, and mixture subgroups, respectively; (**B**) the determination for the number of subgroups via the Delta method of Evanno et al. [55].

To define the subpopulations within the panel of 250 accessions, as described by Pritchard et al. [54], we selected 16,174 of the 43,834 SNPs that were randomly distributed across the 20 soybean chromosomes and had better polymorphisms. STRUCTURE 2.3.4 software was used to calculate delta K (ΔK) (Figure 2B; *k* = 1–10), revealing the existence of three subpopulations (selected *k* = 3) based on ΔK values (Figure 2). All of the high-quality 43,834 SNPs in 250 accessions were used to conduct GWAS for 100-SW. As a result, respectively 66, 76, 45, 55, and 70, QTNs were detected to be associated with 100-SW in five situations (E1–E4 and BLUP) (Tables S1–S6). These represented 218 unique QTNs, of which 156 overlapped with previously reported QTNs, and 62 were found newly in this study; 13–20, 12–24, 8–13, 16–21, 17–25, and 11–22 were identified by the mrMLM, FASTmrMLM, FASTmrEMMA, pLARmEB, pKWmEB, and ISIS EM-BLASSO, respectively, in all the situations (E1–E4 and BLUP) (Table 2 and Tables S1–S6). The LOD values ranged from 3.01 to 18.08, and the proportion of phenotypic variance explained (PVE) by each QTN ranged 0.38–7.88 (%). All these QTNs were distributed on 20 chromosomes, and more than 10 QTNs were found to be located on each of eleven chromosomes, including chromosomes 01, 04–07, 10, 11, 13, 14, 17 and 20 (Tables S1–S6).

Using SL-GWAS (MLM), 1, 1, 3, 5, and 1 QTNs were identified in E1 to E4 and BLUP, respectively (Table S7). Among the eight QTNs, two QTNs (*qcHSW-10-4* and *qHSW-18-4*) were overlapped with those from ML-GWAS.

### 3.3. Stable QTNs for 100-SW in Soybean

**Figure 3:**
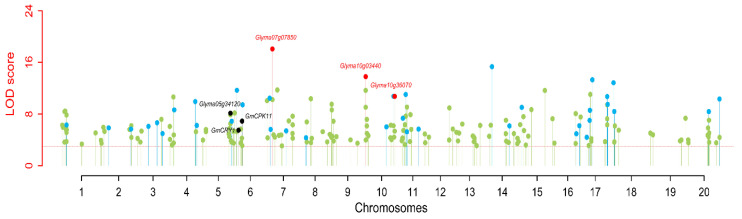
Stable QTNs of soybean 100-seed weight identified in four environments and BLUP values by using six ML-GWAS methods. The black color dots were the stable QTNs near previously reported genes (*Glyma05g34120*, *GmCRY1*, and *GmCPK11*), while the red color dots were the stable QTNs near predicted candidate genes in this study. The sky blue color dots were other stable QTNs, while the light green color dots were remaining significant QTNs.

Two types of QTNs were defined as stable QTNs. One is environmentally-stable QTN (esQTN), which is identified by at least three ML-GWAS methods, while another is methods-stable QTN (msQTN), which is detected in at least three environments/BLUP. In the present study, a total of 43 QTNs (37 QTNs and 6 QTN clusters) were identified as stable QTNs and listed in Table S8. Among the 43 stable QTNs, 36 were msQTNs, 22 were esQTNs, and 15 were common between msQTNs and esQTNs (Figure 3; Table 3 and Table S8). Moreover, eight QTNs were identified in one environment by at least three ML-GWAS methods, while 3 QTNs were detected by one ML-GWAS method in at least three environments/BLUP, and their LOD scores were 3.58–13.31 and 3.19–15.00, respectively (Table S8). Interestingly, three QTNs, *qHSW-4-1*, *qcHSW-7-3* and *qcHSW-10-4*, were identified by six ML-GWAS methods to be associated with 100-SW in all the environments (E1 to E4) and BLUP model, whereas their LOD scores were 3.01–8.64, 4.56–18.08, and 3.26–10.73, respectively, and their PVE values were 1.05–5.34, 2.42–5.91, and 1.37–5.90 (%), respectively (Table 3). Furthermore, seven QTNs *qcHSW-1-1, qHSW-2-2, qHSW-4-2, qHSW-6-1, qcHSW-6-3, qHSW-8-1*, and *qHSW-11-3*, were detected, respectively, by five, six, three, six, five, three and one ML-GWAS methods to be associated with 100-SW in three environments/BLUP model, whereas their LOD scores were 4.71–6.30, 3.14–5.66, 3.34–9.93, 3.50–11.68, 3.13–6.90, 3.17–4.33, and 3.19–5.25, respectively, and their PVE values were 2.93–4.94, 1.06–2.53, 1.11–4.20, 1.61–6.94, 0.97–3.59, 0.68–1.79, and 1.34–2.88 (%), respectively (Table 3). Approximately 33 (76.74%) QTNs were overlapped or located near the genomic region of previously reported QTLs for 100-SW, while 10 (23.26%) were newly identified.

### 3.4. Validation of Stable QTNs for 100-SW in Soybean

The above 43 stable QTNs were used for SW increasing allele analysis in order to validate these stable QTNs. The 100-SW average of accessions having SW increasing alleles was 1.64–20.05 (g) higher than that of the accessions with SW decreasing alleles and 0.3–18.83 (g) higher than that of all the accessions (Table 3 and Table S8). These QTNs showed the significant differences of 100-SW between SW increasing and decreasing alleles at the 0.01 level (Figures S1–S6). For example, for eleven stable QTNs (*qcHSW-1-1, qHSW-2-2, qHSW-4-1, qHSW-4-2, qHSW-4-3, qHSW-6-1, qcHSW-6-3, qcHSW-7-3, qHSW-8-1, qcHSW-10-4*, and *qHSW-11-3*), the 100-SW averages of accessions with SW increasing alleles A, C, C, A, C, G, T, T, C, C, and A significantly increased 5.39–6.34, 3.67–5.49, 34.3–5.16, 4.66–5.37, 3.78–4.14, 3.42–4.24, 3.90–5.26, 2.84–3.24, 2.69–3.59, 2.24–3.62, and 4.96–5.91 (g), respectively, across three environments as compared with those with the corresponding SW decreasing alleles (Figures S1 and S6; Table 3); for seven stable QTNs (*qHSW-7-4, qcHSW-10-1, qHSW-14-2, qHSW-15-1, qHSW-16-1*, and *qHSW-16-2*) newly identified in this study, the 100-SW averages of accessions with SW increasing alleles A, C, A, C, A, and G were significantly higher than those with SW decreasing alleles G, G, G, A, T, and C, respectively (Figures S3–S6; Table S8).

### 3.5. Prediction of the Best Parental Combinations for 100-Seed Weight in Two Directions

The number of SW increasing allele for each stable QTN in 250 accessions ranged from 3 (1.2%) to 225 (90.00%). Among the above 43 stable QTNs, 21 had more than 50% SW increasing alleles in the 250 accessions, while 22 had less than 50% SW increasing alleles in the 250 accessions (Table S9). The number of SW increasing alleles for each accession ranged from 8 (18.60%) to 36 (83.72%). Among the 250 accessions, 69 had more than 22 (50%) SW increasing alleles, while 181 had less than 21 (50%) SW increasing alleles. Interestingly, eight accessions, Yixingwuhuangdou, Yafanzaodou, Nannong 95C-5, Ribendaheidou, Fujiandadou, Quxiandahuangdou, Bayueqing, and Nanchengqingpidadou had 36, 36, 35, 34, 30, 29, 27, and 27 SW increasing alleles, respectively. Six accessions Qinyan 1, Baihuadou, Mayidan, Mingshanhongxingjiroudou, Heibiqing, and Qingcha 1 had 11, 10, 9, 8, 8, and 8 SW increasing alleles, respectively, while these accessions had 30, 28, 33, 30, 25, and 32 SW decreasing alleles, respectively. All the accessions can be used for the soybean breeding program by increasing or decreasing the number of SW increasing alleles in one cultivar. For example, the cross between Yafanzaodou (36 SW increasing alleles) and Ribendaheidou (34 SW increasing alleles) may produce the offspring with 41 SW increasing alleles. Thus, the best five cross combinations of large and small seeds were predicted and listed in Table 4. It should be noted that some parents were repeatedly present in these predicted combinations. For example, Ribendaheidou was used as parent in three cross combinations for larger seed, while Heibiqing, Mayidan, and Qinyan1 were used as parents in at least two combinations for small seeds (Table 4).

### 3.6. Candidate Genes Underlying the Stable QTNs for 100-SW in Soybean

**Figure 4:**
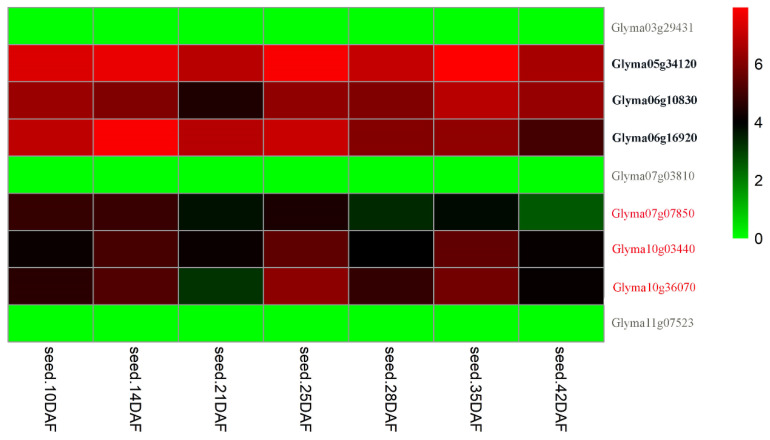
The expressional levels [log_2_(RPKM + 1)] of potential candidate genes associated with seed weight in seven soybean tissues. Among the nine genes, three were previously reported seed-weight genes (bold text) in soybean, three were lowly expressed genes (grey text), and three were newly identified as candidate genes (red text) to be related to seed size/weight and development.

**Figure 5:**
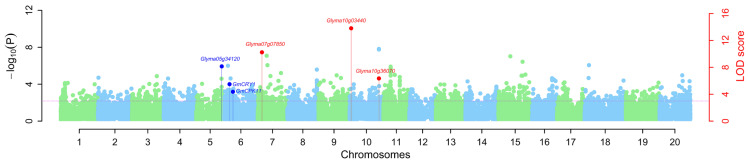
Manhattan plot in the detection of QTNs for 100-seed weight in soybean using multi-locus genome-wide association study approaches. The blue color dots were used to represent the QTNs near previously reported genes (*Glyma05g34120*, *GmCRY1*, and *GmCPK11*), and the red color dots were used to represent the QTNs near predicted candidate genes in this study, whereas light blue and light green color dots were used to indicate the negative log_10_(*p*-value) of each marker on the adjacent chromosomes in the first step of multi-locus approaches.

A search for putative candidate genes resulted in 774 potential candidate genes located between 50 kb–100 kb up- or downstream of the above 43 stable QTNs. Among the 774 genes, 205 exhibited high expression levels at seven seed development stages. Among the 205 genes, 175 were found to have homologs in *Arabidopsis*. Among these homologs, 36 genes were found to be related to seed weight (Table S10). Kyoto encyclopedia of genes and genomes (KEGG, http://kobas.cbi.pku.edu.cn/kobas3) analysis from the above 36 genes indicated that nine genes were involved in eleven seed-weight-related pathways (Table S10). In RNA-seq analysis, twenty-nine genes had two times higher gene expression, as compared with *Glyma03g29431, Glyma04g33610, Glyma06g07940, Glyma07g03810, Glyma07g05260, Glyma11g07523*, and *Glyma20g21082* in seed development stages (Figure S7). Between the above nine and twenty-nine genes, there were six common genes, which were considered as candidate genes in this study (Table 5; Figure 4 and Figure 5). Among these candidate genes, *Glyma05g34120, Glyma06g10830* (*GmCRY1*), and *Glyma06g16920* (*GmCPK11*) had known functions for seed size/weight in soybean (Table 5; Figure 4 bold text), and the others were homologous to the known genes for seed size and seed development in *Arabidopsis*, for example, candidate gene *Glyma07g07850* underlying the stable QTN *qcHSW-7-3* were homologous to *AT4G00710* (*BSK3*), which annotate BR-signaling kinase 3 in *Arabidopsis*. *Glyma10g03440* and *Glyma10g36070* underlying the stable QTNs *qcHSW-10-1* and *qcHSW-10-4* were homologous to, respectively, the *Arabidopsis* genes *AT1G03090* (*MCCA*) and *AT1G35680* (*RPL21*), which are related to seed weight or development (Table 5; Figure 4 and Figure 5).

## 4. Discussion

To dissect the genetic basis of 100-SW and provide SW increasing alleles for molecular breeding in soybean, the 100-SW phenotypes of 250 soybean accessions in four environments were used to be associated with 43,834 SNP markers using seven GWAS approaches in this study. As a result, we obtained some valuable results in two aspects. On one hand, 43 stable QTNs were identified, and showed significant differences of 100-SW between the two alleles (Figure S1–S6; Table 3 and Table S8). Using the above 43 stable QTN information, new cross combinations were predicted (Table 4), and a number of SSR markers were obtained from overlapping and previously published QTLs and comparative genomics analysis (Table 6 and Table S8). Thus, these SSR markers can be used to conduct marker-assisted selection in soybean breeding. Based on the above 43 stable QTNs, on the other hand, multi-omics analyses were used to mine candidate genes. As a result, six candidate genes were obtained in this study. Among the six candidate genes, *Glyma05g34120*, *Glyma06g10830* (*GmCRY1*), and *Glyma06g16920* (*GmCPK11*) were found to be associated with soybean 100-SW, respectively, in Li et al. [7], Du et al. [37], and Aghamirzaie et al. [66], and *Glyma07g07850*, *Glyma10g03440*, and *Glyma10g36070* were new in soybean (Table 5). These new 100-SW-related candidate genes are valuable in soybean molecular biology research.

### 4.1. Comparison of Stable QTNs in This Study with Previously Reported QTLs

Up to now, more than 200 QTLs/QTNs were identified by QTL mapping and/or GWAS to be associated with seed weight in soybean (http://soybase.org/). Thus, it is possible to compare these results with 43 stable QTNs in this study. As a result, 33 stable QTNs were found to be located in the genomic regions of previously reported QTLs underlying seed weight (Table 6). For example, two stable QTNs *qHSW-2-1* and *qHSW-2-2* were located simultaneously in the genomic region of one known QTL SW 49-8 [17], whereas one stable QTN *qcHSW-1-1* was overlapped with previously reported QTL SW 15-2 [70]. Two stable QTNs *qHSW-7-2* and *qHSW-11-1* were found in the genomic region of known QTLs SW 49-15 [17] and SW 37-9 [71], respectively. Interestingly, five stable QTNs *qHSW4-3, qcHSW-6-3, qHSW-10-3, qHSW-17-1, qHSW-20-2* were identified in the genomic region of previously reported QTLs SW 45-3 [72], SW 4-1, SW 25-4 [73], SW 23-1 [74], SW 42-2 [75], and SW 50-16 [76], respectively. In previous study, Teng et al. [17] identified QTL SW 49-10 underlying seed weight, and this QTL was consistent with our two stable QTNs *qHSW-17-5* and *qHSW-17-6* (Table 6). In addition, the QTLs reported in Han et al. [14], Hyten et al. [70], Kato et al. [76], Li et al. [77], Teng et al. [17], Yan et al. [72], and Yao et al. [78] were found as well to be consistent with our stable QTNs in this study. Therefore, 33 stable QTNs were overlapped with previously reported QTLs, indicating the accuracy of our QTN detection. More importantly, 10 stable QTNs (*qHSW-3-1, qHSW-3-2, qHSW-3-3, qHSW-4-1, qHSW-7-4, qcHSW-10-1, qHSW-14-2, qHSW-15-1, qHSW-16-1*, and *qHSW-16-2*) were newly identified in this study.

### 4.2. Reliability of QTNs and Application of SW Increasing Allele in Soybean Breeding

In this study, 218 significant QTNs were identified to be associated with 100-SW in soybean. Among them, a total of 43 QTNs were repeatedly identified in more than three environments and/or methods, and viewed as stable QTNs. Of these stable QTNs, 36 QTNs were identified in at least three environments/BLUP by multiple methods and 22 QTNs were detected by at least three methods in multiple environments. Among them, eight were detected in one environment by at least three ML-GWAS methods, whereas three QTNs were detected by one ML-GWAS method in at least three environments/BLUP (Table 3 and Table S8). The QTNs found across different environments are reliable, i.e., Zhou et al. [8] repeatedly detected 31 QTNs associated with 100-SW in 185 soybean accessions in multiple environments, while Li et al. [7] identified 35 QTNs associated with soybean yield traits in at least three environments. Likewise, the QTNs identified by multiple methods are also reliable when several multi-locus approaches are used to evaluate the same dataset [23]. For example, 58 QTNs associated with embryonic callus-related traits have been detected by at three multi-locus methods in Ma et al. [49], seven QTNs associated with starch pasting properties-relate traits in maize have been identified by more than one method in Xu et al. [89], and all the 56 QTNs associated with seven salt tolerance-related traits have been determined by at least three multi-locus methods in Cui et al. [48].

To verify the reliability of each stable QTN, we divided the 250 accessions into two groups based on their allelic types and compared the mean phenotypic values of both alleles. As a result, forty-three QTNs exhibited significant differences of 100-SW between the two alleles (Figures S1–S6), suggesting the reliability of QTNs identified in this study. More importantly, these SW increasing alleles can be utilized in molecular breeding [7,8,18,25,61].

In this study, the average number of SW increasing alleles per accession was 18.42, indicating the predominance of SW increasing alleles in cultivars after the disappearance of some alleles during artificial or natural selection process. Based on these SW increasing and decreasing alleles, we also predict some parental combinations. In these combinations, the cultivars Ribendaheidou, Mayidan, and Heibiqing are repeatedly present. These predictions might be valuable for the following reasons. Firstly, three selected parents, Yixingwuhuangdou, Quxiandahuangdou, and Nannong 95C-5, were also predicted as parents for seed size traits based on the effects of elite alleles in Niu et al. [18]. Secondly, some selected parents have been widely planted in some areas owing to their high yield, e.g., Fujiandadou, Nannong 95C-5, Yixingwuhuangdou, Quxiandahuang-dou, and Ribendaheidou. The similar idea can be found in rice breeding, e.g., Wang et al. [90] developed a *japonica* cultivar (chromosome segment substitution line) for large grain (>8.5 mm grain length × 3.2 mm grain width) by molecular breeding, demonstrating that elite alleles from different cultivars can be pyramided into a new cultivar.

### 4.3. Candidate Genes Underlying Stable QTNs for Seed Weight

Identification of candidate genes underlying stable QTNs is of great interest for practical plant breeding and is necessary for further gene cloning and functional verification. To date, only a few seed-weight-related genes have been identified based on association mapping in soybean. Based on functional annotations, available literature, comparative genome analysis, KEGG pathways, and gene expression data, the present study mined candidate genes regulating seed size/weight and development in soybeans. Among 774 genes within the physical regions of 43 stable QTNs, therefore, 36 genes were considered as candidate genes to be involved in seed size/weight and development. Among the 36 candidate genes (Table S10), nine were found in KEGG pathway analysis, 29 had significantly higher expressed at seed developmental stages, and there were six common genes between the nine and twenty-nine genes (Table 5; Figure 5). Among the six candidate genes, *Glyma05g34120*, *Glyma06g16920* (*GmCPK11*), and *Glyma06g10830* (*GmCRY1*) have been reported to directly control seed weight in soybean and to have seed size/weight related functions [7,37,66]. Therefore, these candidate genes are very reliable and useful in the improvement of 100-SW in soybean.

The other three candidate genes are newly identified in this study. *Glyma07g07850* is homologous to *BSK3*, one brassinosteroid (BR) biosynthesis or signaling gene in *Arabidopsis*. The *BSK3* gene has a decisive role in the initial steps of the BR signal transduction pathway [91], and mutant *bsk3* has been known to play an important role in seed size [67]. BRs are plant hormones that regulate plant growth and development, and the deficiency of this hormone causes abnormal plant growth and hence yield reduction [92]. Physiological, cellular, and molecular mechanisms influencing plant growth and yield production also indicate the diverse role of BR in plant growth and development [93]. In addition, some factors have been found to affect the soybean 100-SW, such as seed size, hormones (ABA, BRs, GA3, and IAA), enzymes, silique development, cell growth rate, cotyledon cell number, pollen development and cell volume [13,94].

*Glyma10g03440* encodes 3-Methylcrotonyl CoA carboxylase (*MCCA*), which is a nuclear-encoded biotin-localized enzyme and also plays an important role in leucine and isoprenoids catabolism. In *Arabidopsis*, knockout alleles of the *MCCA* gene and metabolite study suggest that *MCCA* mutations block mitochondrial leucine catabolism, which is associated with reduced reproductive growth phenotype, including abnormal flower and silique development [68]. *Glyma10g36070* encodes ribosomal protein L21 (*RPL21*) that is required for chloroplast and pollen development and embryogenesis in *Arabidopsis* [69]. However, these candidate genes require further functional validation/cloning to determine their actual role in seed weight in soybean.

### 4.4. Statistical Power of Multi-Locus GWAS Approaches

In this study, 218 significant QTNs for 100-SW in soybean were detected from six ML-GWAS approaches. These significant QTNs were divided into four groups. In the first group, all the QTNs are both msQTNs and esQTNs. All the QTNs in the second group are esQTNs rather than msQTNs, while all the QTNs in the third group are msQTNs rather than esQTNs. In the last group, all the QTNs are neither esQTNs nor msQTNs. Thus, we summarized their characteristics of the above four groups, such as the number of significant QTNs, the average of absolute effects, LOD score and r^2^, and the proportion of previously reported QTNs in Table 7. As a result, it is easy (the highest proportion of previously reported QTNs) to identify the QTNs in the first group and these QTNs have the largest values for QTN effects, LOD scores and r^2^, while it is relatively difficult (the lowest proportion of previously reported QTNs) to detect the QTNs in the last group and these QTNs have relatively small values for QTN effects, LOD scores, and r^2^. The above results show the advantage of our multi-locus GWAS approaches in detecting small-effect QTNs. The results support our previous recommendation that the QTNs identified by individual approaches or in individual environments are valuable in mining the genes for the trait of interest [23].

In addition, we also found the gap between the trait heritability (83.23–93.70%) and the sum of r^2^ (24.13–35.52%) for all the QTNs identified by each approach in one environment or BLUP. This is the heritability missing in GWAS [23]. The possible reasons are the exclusion of QTN-by-environment and QTN-by-QTN interactions in this study. Thus, it is necessary to develop the methodologies for detecting QTN-by-environment and QTN-by-QTN interactions in the near future.

## 5. Conclusions

In this study, 43 stable QTNs were detected in at least three environments/BLUP and/or by at least three ML-GWAS methods, and they showed significant differences of 100-SW between the two alleles in the GWAS population. Using these SW increasing or decreasing alleles of stable QTNs, the best five cross combinations were predicted in large or small seed directions. Among the 36 potential candidate genes from multi-omics analysis, *Glyma05g34120*, *GmCRY1*, and *GmCPK11* are the known seed-size-related genes in soybean, and *Glyma07g07850*, *Glyma10g03440*, and *Glyma10g36070* were identified to be candidate genes in this study.

## Figures and Tables

**Table 1:** Phenotypic analysis of soybean 100-seed weight in 250 accessions in four environments.

Environment	Mean	Min	Max	SD	CV (%)	Skew	Kur	F_G_	F_E_	h^2^_B_ (%)
**E1**, Nanjing 2014	18.39	6.18	44.53	5.96	32.39	0.95	1.07	25.43 **	18.65 **	93.70
**E2**, Nanjing 2015	19.86	5.28	40.64	5.58	28.08	0.49	0.62			88.51
**E3**, Wuhan 2014	17.98	6.75	37.57	5.07	28.20	0.69	1.09			90.15
**E4**, Wuhan 2015	19.22	8.83	51.19	5.58	29.07	1.03	1.13			83.23

SD: standard deviation; CV: coefficient of variation; Skew: skewness; Kur: kurtosis; F_G_ and F_E_: F values for genotype and environment, respectively; h^2^_B_: broad sense heritability; **: significance at the 0.01 level.

**Table 2:** Summary of QTNs identified in four environments and their BLUP values using six ML-GWAS methods.

Method	E1	E2	E3	E4	BLUP	QTN Effect	LOD Score	r^2^ (%)
Total	66	76	45	55	70	−4.66–4.07	3.01–18.08	0.38–7.88
mrMLM	20	19	15	16	13	−4.66–2.11	3.01–11.72	0.72–6.94
FASTmrMLM	18	22	14	12	24	−2.60–2.31	3.01–13.80	0.49–7.88
FASTmrEMMA	10	10	9	8	13	−3.48–4.07	3.18–12.96	1.02–7.26
pLARmEB	21	18	16	18	20	−3.98–2.54	3.03–15.34	0.38–6.90
pKWmEB	17	25	17	18	20	−2.55–1.88	3.04–12.84	1.04–5.52
ISIS EM-BLASSO	22	18	11	16	22	−4.61–1.73	3.11–18.08	0.66–5.91

E1: Nanjing (2014); E2: Nanjing (2015); E3: Wuhan (2014); E4: Wuhan (2015); r^2^ (%): the proportion of total phenotypic variation explained by each QTN.

**Table 3:** Stable QTNs for soybean 100-seed weight identified in multiple environments and the BLUP model.

QTN ^a^	Position (bp)	Effect	LOD Score	r^2^ (%) ^b^	MAF	Method ^c^	Environment ^d^	SW Increasing Allele	Average 100-Seed Weight				SSR Marker
									SW Increasing Allele	SW Decreasing Allele	Total	Significance ^e^	
***qcHSW-1-1***	Gm01_6389301-6594737	1.36–4.07	4.71–6.30	2.93–4.94	0.09	1–3, 5, 6	E1–E3, BLUP	A	18.34–20.29	12.05–13.95	17.98–19.86	**	BARCSOYSSR_01_0337
***qHSW-2-2***	Gm02_43506875	0.83–2.77	3.14–5.66	1.06–2.53	0.13	1–6	E1, E3, E4, BLUP	C	23.23–24.20	17.91–19.56	18.3919.86	**	BARCSOYSSR_02_1373
*qHSW-3-1*	Gm03_17127049	0.76–1.49	3.86–6.09	0.61–1.67	0.1	2, 4	E1, E2, BLUP	G	22.50–24.89	17.45–17.68	17.98–18.39	**	BARCSOYSSR_03_0519
***qHSW-3-2***	Gm03_29644613	0.54–0.82	3.67–6.65	1.49–2.44	0.33	2, 5, 6	E1, E2, BLUP	T	19.12–19.48	16.05–16.51	17.98–18.39	**	BARCSOYSSR_03_0762
*qHSW-3-3*	Gm03_37435877	−2.55–1.35	3.18–4.98	0.67–3.24	0.02	2, 5	E1, E4, BLUP	C	26.24–27.94	18.20–19.01	18.39–19.22	**	BARCSOYSSR_03_1155
***qHSW-4-1***	Gm04_6703334	−3.33–0.48	3.01–8.64	1.05–5.34	0.2	1–6	E1–E4, BLUP	C	21.37–24.09	17.20–18.93	17.98–19.22	**	BARC-025825-05102
***qHSW-4-2***	Gm04_37026887	0.59–1.71	3.34–9.93	1.11–4.20	0.26	1–3	E1–E3, BLUP	A	19.29–21.24	14.54–16.33	17.98–19.86	**	BARCSOYSSR_04_0940
***qHSW-4-3***	Gm04_39207103	−1.19–0.75	4.09–6.23	1.22–4.75	0.15	1, 2, 4, 5	E1, E2, E4	C	21.50–22.39	17.36–18.61	17.98–19.22	**	BARCSOYSSR_04_1006
*qHSW-5-1*	Gm05_38490635	0.62–1.24	3.29–8.13	1.24–2.94	0.47	2, 3	E1, E3, BLUP	A	19.64–20.73	17.00–18.85	18.39–19.86	*	BARCSOYSSR_05_1241
*qHSW-5-2*	Gm05_40410433	0.68–1.20	5.24–6.88	1.35–3.4	0.22	6	E1, E4, BLUP	A	19.44–20.16	14.29–15.53	18.39–19.22	**	BARC-007964-00162
***qHSW-6-1***	Gm06_5910218	0.71–2.71	3.50–11.68	1.61–6.94	0.4	1–6	E1–E3, BLUP	G	19.38–21.49	15.71–17.24	17.39–19.86	**	BARC-045145-08894
***qcHSW-6-3***	Gm06_13385023-13385940	0.813–3.44	3.13–6.90	0.97–3.59	0.13	2–6	E1–E3, BLUP	T	18.53–20.49	13.87–15.87	18.39–19.86	**	BARCSOYSSR_06_0614
***qHSW-6-4***	Gm06_14086552	0.45–2.37	3.03–9.43	0.63–5.13	0.38	1–6	E1, E2, BLUP	C	19.16–19.99	15.81–16.07	17.98–18.39	**	BARCSOYSSR_06_0759
***qcHSW-7-3***	Gm07_6439407-6463021	0.94–2.25	4.56–18.08	2.42–5.91	0.41	1–6	E1–E4, BLUP	T	19.74–21.58	16.79–18.74	17.98–18.39	**	BARCSOYSSR_07_0334
***qHSW-8-1***	Gm08_10314889	0.46–0.76	3.17–4.33	0.68–1.79	0.28	1, 5, 6	E1, E2, E4, BLUP	C	19.97–21.47	17.27–18.40	17.98–19.22	**	BARC-038631-07266
***qcHSW-10-4***	Gm10_44349893-44469282	0.66–2.34	3.26–10.73	1.37–5.90	0.27	1–6	E1–E4, BLUP	C	20.08–21.88	17.23–19.28	17.98–19.22	**	BARCSOYSSR_10_1419
*qHSW-11-1*	Gm11_5245829	0.50–0.77	3.38–7.34	1.20–2.27	0.39	5, 6	E1, E2, BLUP	G	19.67–20.24	15.62–15.76	17.98–18.39	**	BARC-018099-02516
***qHSW-11-2***	Gm11_9337224	0.55–2.95	3.09–11.03	1.04–5.50	0.32	1–6	E1, E2, BLUP	A	20.10–21.16	16.95–17.06	17.98–18.39	**	BARCSOYSSR_11_0511
*qHSW-11-3*	Gm11_11100801	−1.39–0.75	3.19–5.25	1.34–2.88	0.12	5	E1, E2, E4, BLUP	A	22.35–24.23	17.38–18.54	17.98–19.22	**	BARCSOYSSR_11_0615
*qHSW-14-1*	Gm14_10032197	0.79–1.52	3.77–15.34	1.36–6.90	0.25	4	E1, E2, BLUP	C	19.13–19.75	14.32–14.54	17.98–18.39	**	BARC-052759-11611
***qHSW-17-6***	Gm17_38985524	−3.38–1.16	4.36–9.49	1.79–4.60	0.1	1–6	E3, E4, BLUP	A	23.35–23.90	18.76–18.77	19.22–19.86	**	BARCSOYSSR_17_1474
***qHSW-20-2***	Gm20_45498156	0.76–1.82	3.26–10.33	1.93–5.09	0.37	1–3, 5	E1, E2, BLUP	G	19.20–20.07	16.33–16.63	17.98–18.39	**	BARC-047899-10425

^a^ The stable QTNs were detected in at least three environments/BLUP, while the bold QTNs were identified by at least three methods. “*qc*”: QTN cluster; “*q*”: QTN. ^b^ r^2^ (%): The proportion of total phenotypic variance explained by each QTN. ^c^ 1: mrMLM; 2: FASTmrMLM; 3: FASTmrEMMA; 4: pLARmEB; 5: pKWmEB; 6: ISIS EM-BLASSO. ^d^ E1: Nanjing (2014); E2: Nanjing (2015); E3: Wuhan (2014); E4: Wuhan (2015). ^e^ * and **: the 0.05 and 0.01 levels of significance, respectively. ^f^ SSR markers: located near the stable QTNs and derived from 33,065 SSR markers in the BARCSOYSSR_1.0 database [65].

**Table 4:** The best parental combinations predicted from genome-wide association studies for 100-seed weight in soybean.

Breeding Objective	Predicted Parental Combinations
Large seed	Yafanzaodou × Ribendaheidou
	Nannong 95C-5 × Ribendaheidou
	Quxiandahuangdou × Yixingwuhuangdou
	Bayueqing × Fujiandadou
	Nanchengqingpidadou × Ribendaheidou
Small seed	Heibiqing × Mayidan
	Qinyan 1 × Mayidan
	Qingcha 1× Mayidan
	Qinyan 1 × Heibiqing
	Mingshanhongxingjiroudou × Mayidan

**Table 5:** Predicted potential candidate genes for 100-seed weight near the stable QTNs in soybean.

Genome-Wide Association Study		Soybean Genes		Comparative Genomic Study			KEGG Pathway	Reference
QTN (QTN Cluster)	Position (bp)	Candidate Gene	Position (bp)	Gene Name	Arabidopsis Gene	Functional Annotation		
*qHSW-5-1*	Gm05_38490635	***Glyma05g34120***	Gm05:38540979-38549756			Translation elongation factor EF1A	mRNA surveillance pathway	[7]
*qHSW-6-2*	Gm06_8258824	***Glyma06g10830***	Gm06:8199290-8204935	*GmCRY1*		cryptochrome 1	Circadian rhythm—plant	[37]
*qcHSW-6-3*	Gm06_13385023-13385940	***Glyma06g16920***	Gm06:13300048-13304817	*GmCPK11*		calcium-dependent protein kinase 2	Plant-pathogen interaction	[66]
*qcHSW-7-3*	Gm07_6439407-6463021	*Glyma07g07850*	Gm07:6497229-6504722	*BSK3*	*AT4G00710*	BR-signaling kinase 3	Plant hormone signal transduction	[67]
*qcHSW-10-1*	Gm10_2563422-2566365	*Glyma10g03440*	Gm10:2482369-2489108	*MCCA*	*AT1G03090*	methylcrotonyl-CoA carboxylase alpha chain	Metabolic pathways	[68]
*qcHSW-10-4*	Gm10_44349893-44469282	*Glyma10g36070*	Gm10:44258213-44261525	*RPL21*	*AT1G35680*	Ribosomal protein L21	Ribosome	[69]

The bold text candidate genes were previously reported in soybean and the remaining newly identified in this study.

**Table 6:** Stable QTNs of soybean 100-seed weight in this study that are reported in previous studies.

QTN or QTN Cluster in This Study		Previously Reported QTLs			QTN or QTN Cluster in This Study		Previously Reported QTLs		
Name	Position (bp)	Name	Marker Associated	Reference	Name	Position (bp)	Name	Marker Associated	Reference
*qcHSW-1-1*	Gm01_6389301-6594737	SW 15-2	Sat_305-Satt531	[70]	*qcHSW-10-4*	Gm10_44349893-44469282	SW 34-8	BARC-028651-05984-Satt479	[14]
*qHSW-2-1*	Gm02_11278769	SW 49-8	Satt172-Satt157	[17]	*qHSW-11-1*	Gm11_5245829	SW 37-9	Sat_149-BARC-029533-06211	[71]
*qHSW-2-2*	Gm02_43506875	SW 49-8	Satt172-Satt157	[17]	*qHSW-11-2*	Gm11_9337224	SW 37-9	Sat_149-BARC-029533-06211	[71]
*qHSW-4-2*	Gm04_37026887	SW 45-3	Sat_042-Sat_322	[72]	*qHSW-11-3*	Gm11_11100801	SW 11-1	BARC-059851-16137-BARC-016279-02316	[79]
*qHSW-4-3*	Gm04_39207103	SW 45-3	Sat_042-Sat_322	[72]	*qHSW-11-4*	Gm11_27803417	SW 10-3	Satt415-BARC-041167-07925	[80]
*qHSW-5-1*	Gm05_38490635	SW 10-1	BARC-060051-16321-BARC-045267-08918	[80]	*qHSW-14-1*	Gm14_10032197	SW 23-1	Satt601- BARC-059265-15700	[81]
*qHSW-5-2*	Gm05_40410433	SW 36-10	BARC-029873-06450-BARC-027778-06658	[14]	*qHSW-17-1*	Gm17_8760885	SW 42-2	Satt154- BARC-058841-15463	[75]
*qHSW-6-1*	Gm06_5910218	cqSW-008	Satt457-BARC-059997-16280	[82]	*qHSW-17-2*	Gm17_12908030	SW 43-2	Satt389-Satt447	[83]
*qHSW-6-2*	Gm06_8258824	SW 33-1	Sat_153-Satt291	[84]	*qHSW-17-3*	Gm17_13325606	SW 43-2	Satt389-Satt447	[83]
*qcHSW-6-3*	Gm06_13385023-13385940	SW 4-1	Sat_246-Satt640	[73]	*qHSW-17-4*	Gm17_16770188	SW 47-2	Satt256-Satt458	[77]
*qHSW-6-4*	Gm06_14086552	SW 16-1	Sat_238-BARC-014491-01561	[85]	*qHSW-17-5*	Gm17_38229512	SW 49-10	BARC-013709-01242-BARC-056481-14397	[17]
*qHSW-7-1*	Gm07_2535953	SW 7-6	Sat_316-Satt201	[86]	*qHSW-17-6*	Gm17_38985524	SW 49-10	BARC-013709-01242-BARC-056481-14397	[17]
*qHSW-7-2*	Gm07_3954121	SW 49-15	Satt323-Satt150	[17]	*qHSW-18-1*	Gm18_5591131	SW 50-4	Satt115-Sat_315	[76]
*qcHSW-7-3*	Gm07_6439407-6463021	SW 45-4	BARC-039383-07310-Satt567	[72]	*qHSW-18-2*	Gm18_6705051	SW/p 6-5	Sat_308-Satt324	[78]
*qHSW-8-1*	Gm08_10314889	SW 34-10	Satt424-Satt390	[14]	*qHSW-20-1*	Gm20_30017454	cqSW-003	BARC-041129-07912-Satt127	[87]
*qcHSW-10-2*	Gm10_32393792-32584066	SW 53-1	BARC-064941-19017-BARC-051153-11022	[88]	*qHSW-20-2*	Gm20_45498156	SW 50-16	Sct_189-Satt623	[76]
*qHSW-10-3*	Gm10_42750933	SW 25-4	BARC-037165-06725-Satt173	[74]					

”*q*”: QTN; “*qc*”: QTN cluster; SW: seed weight; SW/p: seed weight per plant.

**Table 7:** Comparison of four kinds of QTNs for soybean 100-seed weight in this study.

Group	No. of QTNs	Absolute Effect	LOD Score	r^2^ (%)	% Known QTNs
1 (Both esQTN and msQTN)	15	1.56 ± 1.10	6.29 ± 3.65	2.99 ± 1.94	86.67
2 (esQTN rather than msQTN)	7	1.12 ± 0.54	5.70 ± 3.22	2.21 ± 1.63	71.42
3 (msQTN rather than esQTN)	21	1.35 ± 0.87	6.38 ± 3.10	2.86 ± 1.71	66.67
4 (Neither esQTN nor msQTN)	14	1.28 ± 1.01	5.43 ± 2.16	2.35 ± 1.46	50.00

esQTN: the QTN identified in at least three environments/BLUP; msQTN: the QTN detected by at least three methods. r^2^ (%): the proportion of phenotypic variance explained by each QTN; % known QTNs: the percent of previously reported QTNs in all the QTNs detected in this group.

## Supplementary Materials

The following are available online at https://www.mdpi.com/2073-4425/11/7/714/s1, Figures S1 to S6: Phenotypic differences of 100-seed weight between accessions carrying different alleles of each QTN. Figure S1: these QTNs include *qcHSW-1-1* (A), *qHSW-2-1* (B), *qHSW-2-2* (C), *qHSW-3-1* (D), *qHSW-3-2* (E), *qHSW-3-3* (F), *qHSW-4-1* (G), and *qHSW-4-2* (H). Figure S2: these QTNs include *qHSW-4-3* (A), *qHSW-5-1* (B), *qHSW-5-2* (C), *qHSW-6-1* (D), *qHSW-6-2* (E), *qcHSW-6-3* (F), and *qHSW-6-4* (G). Figure S3: these QTNs include *qHSW-7-1* (A), *qHSW-7-2* (B), *qcHSW-7-3* (C), *qHSW-7-4* (D), *qHSW-8-1* (E), *qcHSW-10-1* (F), *qcHSW-10-2* (G), and *qHSW-10-3* (H). Figure S4: these QTNs include *qcHSW-10-4* (A), *qHSW-11-1* (B), *qHSW-11-2* (C), *qHSW-11-3* (D), *qHSW-11-4* (E), *qHSW-14-1* (F), *qHSW-14-2* (G). Figure S5: these QTNs include *qHSW-16-1* (A)*, qHSW-16-2* (B)*, qHSW-17-2* (C)*, qHSW-17-1* (D)*, qHSW-17-3* (E)*, qHSW-17-4* (F)*, qHSW-17-5* (G), and *qHSW-17-6* (H). Figure S6: these QTNs include *qHSW-18-1* (A), *qHSW-18-2* (B), *qHSW-20-1* (C), and *qHSW-20-2* (D). * and **: the significances at the 0.05 and 0.01 levels, respectively, using student’s t-test. The error bars represent standard deviation. E1: Nanjing (2014); E2: Nanjing (2015); E3: Wuhan (2014); E4: Wuhan (2015). Figure S7: The expressional levels [log_2_(RPKM + 1)] of candidate genes associated with seed weight in seven soybean tissues. Table S1: Significant QTNs for 100-seed weight detected in four environments and BLUP model using mrMLM (S1). Table S2: Significant QTNs for 100-seed weight detected in four environments and BLUP model by using FASTmrMLM. Table S3: Significant QTNs for 100-seed weight detected in four environments and BLUP model by using FASTmrEMMA. Table S4: Significant QTNs for 100-seed weight detected in four environments and BLUP model by using pLARmEB. Table S5: Significant QTNs for 100-seed weight detected in four environments and BLUP model by using pKWmEB. Table S6: Significant QTNs for 100-seed weight detected in four environments and BLUP model by using ISIS EM-BLASSO. Table S7: Significant QTNs for 100-seed weight detected in four environments and BLUP model by using MLM. Table S8. Stable QTNs of soybean 100-seed weight identified in multiple environments and/or by multiple methods. Table S9: Distribution of SW increasing alleles in stable QTNs among 250 soybean accessions. Table S10. Predicted candidate genes for seed weight near the stable QTNs in soybean.

## References

[1] Lam H.M., Xu X., Liu X., Chen W., Yang G., Wong F.L., Li M.W., He W., Qin N., Wang B. (2010). Resequencing of 31 wild and cultivated soybean genomes identifies patterns of genetic diversity and selection. Nat. Genet..

[2] Xu Y., Li H.N., Li G.J., Wang X., Cheng L.G., Zhang Y.M. (2011). Mapping quantitative trait loci for seed size traits in soybean (*Glycine max* L. Merr.). Theor. Appl. Genet..

[3] Liu K.S. (2008). Food Use of Whole Soybeans. Soybeans: Chemistry, Production, Processing, and Utilization.

[4] Chen Y., Nelson R.L. (2004). Genetic Variation and Relationships among Cultivated, Wild, and Semiwild Soybean. Crop Sci..

[5] Nawaz M.A., Yang S.H., Chung G., Oscar G. (2018). Wild Soybeans: An Opportunistic Resource for Soybean Improvement. Rediscovery of Landraces as a Resource for the Future.

[6] Nawaz M.A., Rehman H.M., Baloch F.S., Ijaz B., Ali M.A., Khan I.A., Lee J.D., Chung G., Yang S.H. (2017). Genome and transcriptome-wide analyses of cellulose synthase gene superfamily in soybean. J. Plant Physiol..

[7] Li X., Zhang X., Zhu L., Bu Y., Wang X., Zhang X., Zhou Y., Wang X., Guo N., Qiu L. (2019). Genome-wide association study of four yield-related traits at the R6 stage in soybean. BMC Genet..

[8] Zhao X., Dong H., Chang H., Zhao J., Teng W., Qiu L., Li W., Han Y. (2019). Genome wide association mapping and candidate gene analysis for hundred seed weight in soybean [*Glycine max* (L.) Merrill]. BMC Genom..

[9] Zhou Z., Lakhssassi N., Cullen M.A., El Baz A., Vuong T.D., Nguyen H.T., Meksem K. (2019). Assessment of phenotypic variations and correlation among seed composition traits in mutagenized soybean populations. Genes.

[10] Russell J.S. (1988). Soybeans: Improvement, production, and uses. Field Crops Res..

[11] Agarwal M., Shrivastava N., Padh H. (2008). Advances in molecular marker techniques and their applications in plant sciences. Plant Cell Rep..

[12] Hoeck J.A., Fehr W.R., Shoemaker R.C., Welke G.A., Johnson S.L., Cianzio S.R. (2003). Molecular marker analysis of seed size in soybean. Crop Sci..

[13] Mian M.A.R., Bailey M.A., Tamulonis J.P., Shipe E.R., Carter T.E., Parrott W.A., Ashley D.A., Hussey R.S., Boerma H.R. (1996). Molecular markers associated with seed weight in two soybean populations. Theor. Appl. Genet..

[14] Han Y., Li D., Zhu D., Li H., Li X., Teng W., Li W. (2012). QTL analysis of soybean seed weight across multi-genetic backgrounds and environments. Theor. Appl. Genet..

[15] Xie F.T., Niu Y., Zhang J., Bu S.H., Zhang H.Z., Geng Q.C., Feng J.Y., Zhang Y.M. (2014). Fine mapping of quantitative trait loci for seed size traits in soybean. Mol. Breed..

[16] Mansur L.M., Orf J.H., Chase K., Jarvik T., Cregan P.B., Lark K.G. (1996). Genetic mapping of agronomic traits using recombinant inbred lines of soybean. Crop Sci..

[17] Teng W., Han Y., Du Y., Sun D., Zhang Z., Qiu L., Sun G., Li W. (2009). QTL analyses of seed weight during the development of soybean (*Glycine max* L. Merr.). Heredity.

[18] Niu Y., Xu Y., Liu X.F., Yang S.X., Wei S.P., Xie F.T., Zhang Y.M. (2013). Association mapping for seed size and shape traits in soybean cultivars. Mol. Breed..

[19] Thomson M.J. (2014). High-Throughput SNP Genotyping to Accelerate Crop Improvement. Plant Breed. Biotechnol..

[20] Wen Y.J., Zhang H., Ni Y.L., Huang B., Zhang J., Feng J.Y., Wang S.B., Dunwell J.M., Zhang Y.M., Wu R. (2018). Methodological implementation of mixed linear models in multi-locus genome-wide association studies. Brief. Bioinform..

[21] Ron M., Weller J.I. (2007). From QTL to QTN identification in livestock—Winning by points rather than knock-out: A review. Anim. Genet..

[22] Wang S.B., Feng J.Y., Ren W.L., Huang B., Zhou L., Wen Y.J., Zhang J., Dunwell J.M., Xu S., Zhang Y.M. (2016). Improving power and accuracy of genome-wide association studies via a multi-locus mixed linear model methodology. Sci. Rep..

[23] Zhang Y.M., Jia Z., Dunwell J.M. (2019). Editorial: The applications of new multi-locus GWAS methodologies in the genetic dissection of complex traits. Front. Plant Sci..

[24] Hao D., Cheng H., Yin Z., Cui S., Zhang D., Wang H., Yu D. (2012). Identification of single nucleotide polymorphisms and haplotypes associated with yield and yield components in soybean (*Glycine max*) landraces across multiple environments. Theor. Appl. Genet..

[25] Zhou L., Wang S.B., Jian J., Geng Q.C., Wen J., Song Q., Wu Z., Li G.J., Liu Y.Q., Dunwell J.M. (2015). Identification of domestication-related loci associated with flowering time and seed size in soybean with the RAD-seq genotyping method. Sci. Rep..

[26] Zhang J., Song Q., Cregan P.B., Jiang G.L. (2016). Genome-wide association study, genomic prediction and marker-assisted selection for seed weight in soybean (*Glycine max*). Theor. Appl. Genet..

[27] Yan L., Hofmann N., Li S., Ferreira M.E., Song B., Jiang G., Ren S., Quigley C., Fickus E., Cregan P. (2017). Identification of QTL with large effect on seed weight in a selective population of soybean with genome-wide association and fixation index analyses. BMC Genom..

[28] Jing Y., Zhao X., Wang J., Teng W., Qiu L., Han Y., Li W. (2018). Identification of the genomic region underlying seed weight per plant in soybean (*Glycine max* L. Merr.) via high-throughput single-nucleotide polymorphisms and a genome-wide association study. Front. Plant Sci..

[29] Assefa T., Otyama P.I., Brown A.V., Kalberer S.R., Kulkarni R.S., Cannon S.B. (2019). Genome-wide associations and epistatic interactions for internode number, plant height, seed weight and seed yield in soybean. BMC Genom..

[30] Hu D., Zhang H., Du Q., Hu Z., Yang Z., Li X., Wang J., Huang F., Yu D., Wang H. (2020). Genetic dissection of yield-related traits via genome-wide association analysis across multiple environments in wild soybean (*Glycine soja* Sieb. and Zucc.). Planta.

[31] Jofuku K.D., Omidyar P.K., Gee Z., Okamuro J.K. (2005). Control of seed mass and seed yield by the floral homeotic gene APETALA2. Proc. Natl. Acad. Sci. USA.

[32] Ohto M.A., Fischer R.L., Goldberg R.B., Nakamura K., Harada J.J. (2005). Control of seed mass by APETALA2. Proc. Natl. Acad. Sci. USA.

[33] Schruff M.C., Spielman M., Tiwari S., Adams S., Fenby N., Scott R.J. (2006). The AUXIN RESPONSE FACTOR 2 gene of Arabidopsis links auxin signalling, cell division, and the size of seeds and other organs. Development.

[34] Zhou Y., Zhang X., Kang X., Zhao X., Zhang X., Ni M. (2009). Short Hypocotyl Under Blue1 associates with Miniseed3 and Haiku2 promoters in vivo to regulate Arabidopsis seed development. Plant Cell.

[35] Sun X., Shantharaj D., Kang X., Ni M. (2010). Transcriptional and hormonal signaling control of Arabidopsis seed development. Curr. Opin. Plant Biol..

[36] Lu X., Li Q.T., Xiong Q., Li W., Bi Y.D., Lai Y.C., Liu X.L., Man W.Q., Zhang W.K., Ma B. (2016). The transcriptomic signature of developing soybean seeds reveals the genetic basis of seed trait adaptation during domestication. Plant J..

[37] Du J., Wang S., He C., Zhou B., Ruan Y.L., Shou H. (2017). Identification of regulatory networks and hub genes controlling soybean seed set and size using RNA sequencing analysis. J. Exp. Bot..

[38] Lu X., Xiong Q., Cheng T., Li Q.T., Liu X.L., Bi Y.D., Li W., Zhang W.K., Ma B., Lai Y.C. (2017). A PP2C-1 allele underlying a quantitative trait locus enhances soybean 100-seed weight. Mol. Plant.

[39] Wang J., Chu S., Zhang H., Zhu Y., Cheng H., Yu D. (2016). Development and application of a novel genome-wide SNP array reveals domestication history in soybean. Sci. Rep..

[40] Gu Y., Li W., Jiang H., Wang Y., Gao H., Liu M., Chen Q., Lai Y., He C. (2017). Differential expression of a WRKY gene between wild and cultivated soybeans correlates to seed size. J. Exp. Bot..

[41] Yang Z., Xin D., Liu C., Jiang H., Han X., Sun Y., Qi Z., Hu G., Chen Q. (2013). Identification of QTLs for seed and pod traits in soybean and analysis for additive effects and epistatic effects of QTLs among multiple environments. Mol. Genet. Genom..

[42] Di S., Yan F., Rodas F.R., Rodriguez T.O., Murai Y., Iwashina T., Sugawara S., Mori T., Nakabayashi R., Yonekura-Sakakibara K. (2015). Linkage mapping, molecular cloning and functional analysis of soybean gene *Fg3* encoding flavonol 3-O-glucoside/galactoside (1 → 2) glucosyltransferase. BMC Plant Biol..

[43] Zhang Y., He J., Wang Y., Xing G., Zhao J., Li Y., Yang S., Palmer R.G., Zhao T., Gai J. (2015). Establishment of a 100-seed weight quantitative trait locus-allele matrix of the germplasm population for optimal recombination design in soybean breeding programmes. J. Exp. Bot..

[44] Yang H., Wang W., He Q., Xiang S., Tian D., Zhao T., Gai J. (2019). Identifying a wild allele conferring small seed size, high protein content and low oil content using chromosome segment substitution lines in soybean. Theor. Appl. Genet..

[45] Zhou Z., Jiang Y., Wang Z., Gou Z., Lyu J., Li W., Yu Y., Shu L., Zhao Y., Ma Y. (2015). Resequencing 302 wild and cultivated accessions identifies genes related to domestication and improvement in soybean. Nat. Biotechnol..

[46] Zhou L., Luo L., Zuo J.-F., Yang L., Zhang L., Guang X., Niu Y., Jian J., Geng Q.-C., Liang L. (2016). Identification and validation of candidate genes associated with domesticated and improved traits in soybean. Plant Genome.

[47] Fang C., Ma Y., Wu S., Liu Z., Wang Z., Yang R., Hu G., Zhou Z., Yu H., Zhang M. (2017). Genome-wide association studies dissect the genetic networks underlying agronomical traits in soybean. Genome Biol..

[48] Cui Y., Zhang F., Zhou Y. (2018). The application of multi-locus GWAS for the detection of salt-tolerance loci in rice. Front. Plant Sci..

[49] Ma L., Liu M., Yan Y., Qing C., Zhang X., Zhang Y., Long Y., Wang L., Pan L., Zou C. (2018). Genetic dissection of maize embryonic callus regenerative capacity using multi-locus genome-wide association studies. Front. Plant Sci..

[50] Zhang Y.M., Jia Z., Dunwell J.M. (2019). The Applications of New Multi-Locus GWAS Methodologies in the Genetic Dissection of Complex Traits.

[51] Bates D., Mächler M., Bolker B.M., Walker S.C. (2015). Fitting linear mixed-effects models using lme4. J. Stat. Softw..

[52] Xu S. (2013). Mapping quantitative trait loci by controlling polygenic background effects. Genetics.

[53] Ryoo H., Lee C. (2014). Underestimation of heritability using a mixed model with a polygenic covariance structure in a genome-wide association study for complex traits. Eur. J. Hum. Genet..

[54] Pritchard J.K., Stephens M., Donnelly P. (2000). Inference of population structure using multilocus genotype data. Genetics.

[55] Evanno G., Regnaut S., Goudet J. (2005). Detecting the number of clusters of individuals using the software Structure: A simulation study. Mol. Ecol..

[56] Earl D.A., vonHoldt B.M. (2012). STRUCTURE HARVESTER: A website and program for visualizing STRUCTURE output and implementing the Evanno method. Conserv. Genet. Resour..

[57] Zhang J., Feng J.Y., Ni Y.L., Wen Y.J., Niu Y., Tamba C.L., Yue C., Song Q., Zhang Y.M. (2017). PLARmEB: Integration of least angle regression with empirical Bayes for multilocus genome-wide association studies. Heredity.

[58] Tamba C.L., Ni Y.L., Zhang Y.M. (2017). Iterative sure independence screening EM-Bayesian LASSO algorithm for multi-locus genome-wide association studies. PLoS Comput. Biol..

[59] Tamba C.L., Zhang Y.M. (2018). A fast mrMLM algorithm for multi-locus genome-wide association studies. bioRxiv.

[60] Ren W.L., Wen Y.J., Dunwell J.M., Zhang Y.M. (2018). PKWmEB: Integration of Kruskal-Wallis test with empirical Bayes under polygenic background control for multi-locus genome-wide association study. Heredity.

[61] Li D., Zhao X., Han Y., Li W., Xie F. (2019). Genome-wide association mapping for seed protein and oil contents using a large panel of soybean accessions. Genomics.

[62] Jones S.I., Vodkin L.O. (2013). Using RNA-seq to profile soybean seed development from fertilization to maturity. PLoS ONE.

[63] Severin A.J., Woody J.L., Bolon Y.T., Joseph B., Diers B.W., Farmer A.D., Muehlbauer G.J., Nelson R.T., Grant D., Specht J.E. (2010). RNA-seq Atlas of *Glycine max*: A guide to the soybean transcriptome. BMC Plant Biol..

[64] Xie C., Mao X., Huang J., Ding Y., Wu J., Dong S., Kong L., Gao G., Li C.Y., Wei L. (2011). KOBAS 2.0: A web server for annotation and identification of enriched pathways and diseases. Nucleic Acids Res..

[65] Song Q., Jia G., Zhu Y., Grant D., Nelson R.T., Hwang E.Y., Hyten D.L., Cregan P.B. (2010). Abundance of SSR motifs and development of candidate polymorphic SSR markers (BARCSOYSSR_1.0) in soybean. Crop Sci..

[66] Aghamirzaie D., Batra D., Heath L.S., Schneider A., Grene R., Collakova E. (2015). Transcriptome-wide functional characterization reveals novel relationships among differentially expressed transcripts in developing soybean embryos. BMC Genom..

[67] Sreeramulu S., Mostizky Y., Sunitha S., Shani E., Nahum H., Salomon D., Hayun L.B., Gruetter C., Rauh D., Ori N. (2013). BSKs are partially redundant positive regulators of brassinosteroid signaling in *Arabidopsis*. Plant J..

[68] Ding G., Che P., Ilarslan H., Wurtele E.S., Nikolau B.J. (2012). Genetic dissection of methylcrotonyl CoA carboxylase indicates a complex role for mitochondrial leucine catabolism during seed development and germination. Plant J..

[69] Yin T., Pan G., Liu H., Wu J., Li Y., Zhao Z., Fu T., Zhou Y. (2012). The chloroplast ribosomal protein L21 gene is essential for plastid development and embryogenesis in *Arabidopsis*. Planta.

[70] Hyten D.L., Pantalone V.R., Sams C.E., Saxton A.M., Landau-Ellis D., Stefaniak T.R., Schmidt M.E. (2004). Seed quality QTL in a prominent soybean population. Theor. Appl. Genet..

[71] Sun Y.N., Pan J.B., Shi X.L., Du X.Y., Wu Q., Qi Z.M., Jiang H.W., Xin D.W., Liu C.Y., Hu G.H. (2012). Multi-environment mapping and meta-analysis of 100-seed weight in soybean. Mol. Biol. Rep..

[72] Yan L., Li Y.H., Yang C.Y., Ren S.X., Chang R.Z., Zhang M.C., Qiu L.J. (2014). Identification and validation of an over-dominant QTL controlling soybean seed weight using populations derived from *Glycine max* × *Glycine soja*. Plant Breed..

[73] Maughan P.J., Saghai Maroof M.A., Buss G.R. (1996). Molecular-marker analysis of seed-weight: Genomic locations, gene action, and evidence for orthologous evolution among three legume species. Theor. Appl. Genet..

[74] Chen Q.S., Zhang Z.C., Liu C.Y., Xin D.W., Qiu H.M., Shan D.P., Shan C.Y., Hu G.H. (2007). QTL analysis of major agronomic traits in soybean. Agric. Sci. China.

[75] Wang Y., Lu J., Chen S., Shu L., Palmer R.G., Xing G., Li Y., Yang S., Yu D., Zhao T. (2014). Exploration of presence/absence variation and corresponding polymorphic markers in soybean genome. J. Integr. Plant Biol..

[76] Kato S., Sayama T., Fujii K., Yumoto S., Kono Y., Hwang T.Y., Kikuchi A., Takada Y., Tanaka Y., Shiraiwa T. (2014). A major and stable QTL associated with seed weight in soybean across multiple environments and genetic backgrounds. Theor. Appl. Genet..

[77] Li D., Sun M., Han Y., Teng W., Li W. (2010). Identification of QTL underlying soluble pigment content in soybean stems related to resistance to soybean white mold (*Sclerotinia sclerotiorum*). Euphytica.

[78] Yao D., Liu Z.Z., Zhang J., Liu S.Y., Qu J., Guan S.Y., Pan L.D., Wang D., Liu J.W., Wang P.W. (2015). Analysis of quantitative trait loci for main plant traits in soybean. Genet. Mol. Res..

[79] Lee S.H., Park K.Y., Lee H.S., Park E.H., Boerma H.R. (2001). Genetic mapping of QTLs conditioning soybean sprout yield and quality. Theor. Appl. Genet..

[80] Specht J.E., Chase K., Macrander M., Graef G.L., Chung J., Markwell J.P., Germann M., Orf J.H., Lark K.G. (2001). Soybean response to water: A QTL analysis of drought tolerance. Crop Sci..

[81] Li W., Zheng D., Van K., Lee S. (2008). QTL mapping for major agronomic traits across two years in soybean (*Glycine max* L. Merr.). J. Crop Sci. Biotech..

[82] Pathan S.M., Vuong T., Clark K., Lee J.D., Grover Shannon J., Roberts C.A., Ellersieck M.R., Burton J.W., Cregan P.B., Hyten D.L. (2013). Genetic mapping and confirmation of quantitative trait loci for seed protein and oil contents and seed weight in soybean. Crop Sci..

[83] Kuroda Y., Kaga A., Tomooka N., Yano H., Takada Y., Kato S., Vaughan D. (2013). QTL affecting fitness of hybrids between wild and cultivated soybeans in experimental fields. Ecol. Evol..

[84] Moongkanna J., Nakasathien S., Novitzky W.P., Kwanyuen P., Sinchaisri P., Srinives P. (2011). SSR markers linking to seed traits and total oil content in soybean. Thai J. Agric. Sci..

[85] Funatsuki H., Kawaguchi K., Matsuba S., Sato Y., Ishimoto M. (2005). Mapping of QTL associated with chilling tolerance during reproductive growth in soybean. Theor. Appl. Genet..

[86] Orf J.H., Chase K., Jarvik T., Mansur L.M., Cregan P.B., Adler F.R., Lark K.G. (1999). Genetics of soybean agronomic traits: I. Comparison of three related recombinant inbred populations. Crop Sci..

[87] Nichols D.M., Glover K.D., Carlson S.R., Specht J.E., Diers B.W. (2006). Fine mapping of a seed protein QTL on soybean linkage group I and its correlated effects on agronomic traits. Crop Sci..

[88] Kastoori R.R., Jedlicka J., Graef G.L., Waters B.M. (2014). Identification of new QTLs for seed mineral, cysteine, and methionine concentrations in soybean [*Glycine max* (L.) Merr.]. Mol. Breed..

[89] Xu Y., Yang T., Zhou Y., Yin S., Li P., Liu J., Xu S., Yang Z., Xu C. (2018). Genome-wide association mapping of starch pasting properties in maize using single-locus and multi-locus models. Front. Plant Sci..

[90] Wang J., Wan X., Crossa J., Crouch J., Weng J., Zhai H., Wan J. (2006). QTL mapping of grain length in rice (*Oryza sativa* L.) using chromosome segment substitution lines. Genet. Res..

[91] Clouse S.D. (2011). Brassinosteroid signal transduction: From receptor kinase activation to transcriptional networks regulating plant development. Plant Cell.

[92] Ohnishi T., Szatmari A.M., Watanabe B., Fujita S., Bancos S., Koncz C., Lafos M., Shibata K., Yokota T., Sakata K. (2006). C-23 hydroxylation by *Arabidopsis* CYP90C1 and CYP90D1 reveals a novel shortcut in brassinosteroid biosynthesis. Plant Cell.

[93] Yang C.J., Zhang C., Lu Y.N., Jin J.Q., Wang X.L. (2011). The mechanisms of brassinosteroids’ action: From signal transduction to plant development. Mol. Plant.

[94] Hirshfield K., Flannery R., Daie J. (1993). Cotyledon cell number and cell size in relation to seed size and seed yield of soybean. Plant Physiol. Biochem..

