# Supplementary material for: Identification of QTNs and Their Candidate Genes for 100-Seed Weight in Soybean (Glycine max L.) Using Multi-Locus Genome-Wide Association Studies
Source: Genes (Basel). 2020 Jun 27;11(7):714. doi: 10.3390/genes11070714 (PMC7397327; doi:10.3390/genes11070714)
Supplement: Supplementary file 1 [file genes-11-00714-s001.pdf]

Supplemental materials

Identification of QTNs and their candidate genes for 100-seed weight in soybean (*Glycine max* L.) using multi-locus genome-wide association studies

Muhammad Ikram[1], Xu Han[1], Jian-Fang Zuo[1], Jian Song[2], Chun-Yu Han[1], Ya-Wen Zhang[1], Yuan-Ming Zhang[1,*]

1 Crop Information Center, College of Plant Science and Technology, Huazhong Agricultural University, Wuhan 430070, China

2 College of Agriculture, Nanjing Agricultural University, Nanjing 210095, China

* Correspondence: soyzhang@mail.hzau.edu.cn (Y.M.Z.).

Main contents

Figures S1-S7

Tables S1-S10

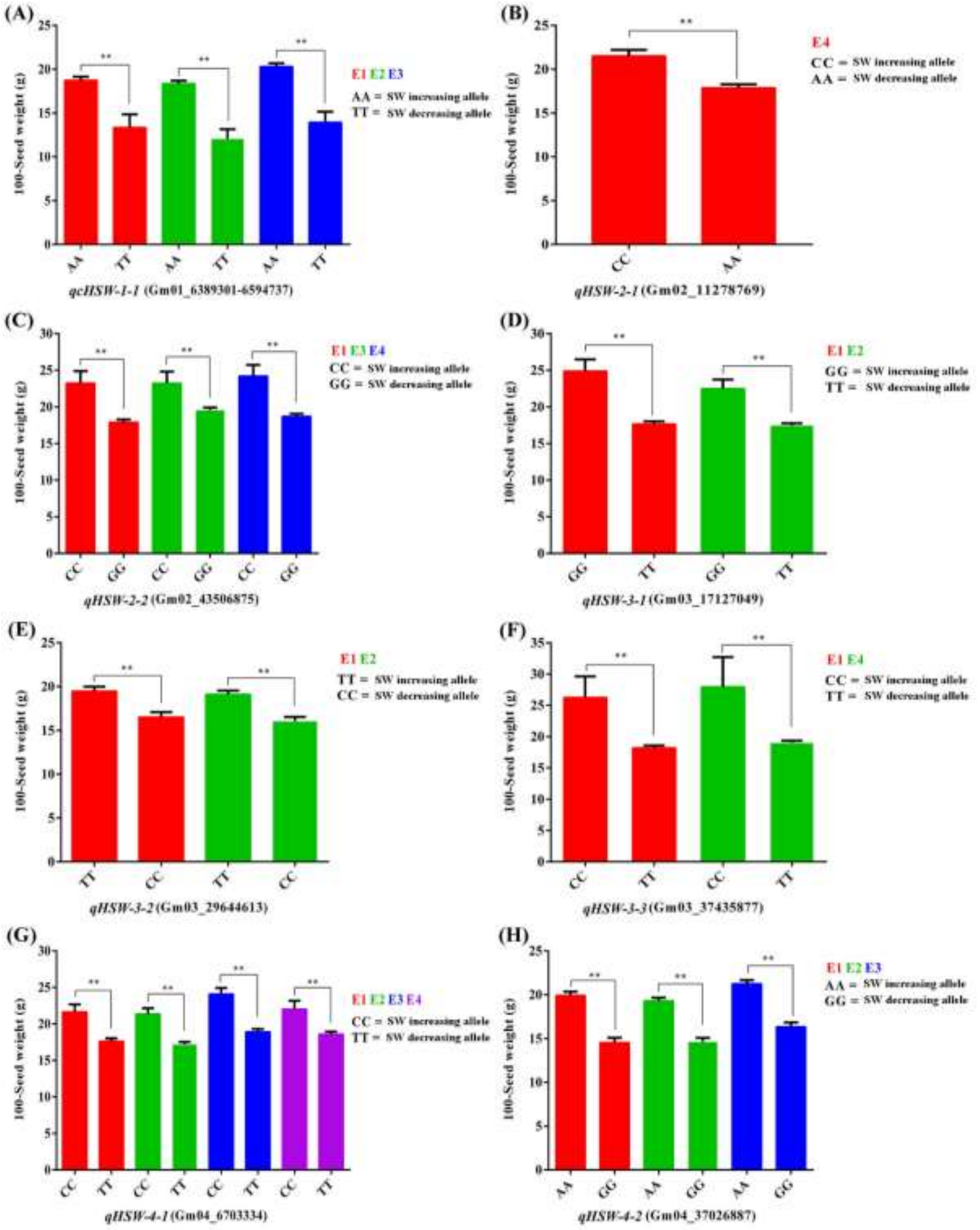

Figure S1: Phenotypic differences of 100-seed weight between accessions carrying different alleles of each QTN. These QTNs include *qcHSW-1-1* (**A**), *qHSW-2-1* (**B**), *qHSW-2-2* (**C**), *qHSW-3-1* (**D**), *qHSW-3-2* (**E**), *qHSW-3-3* (**F**), *qHSW-4-1* (**G**), and *qHSW-4-2* (**H**). * and **: the significances at the 0.05 and 0.01 levels, respectively, using student's t-test. The error bars represent standard deviation. E1: Nanjing (2014); E2: Nanjing (2015); E3: Wuhan (2014); E4: Wuhan (2015).

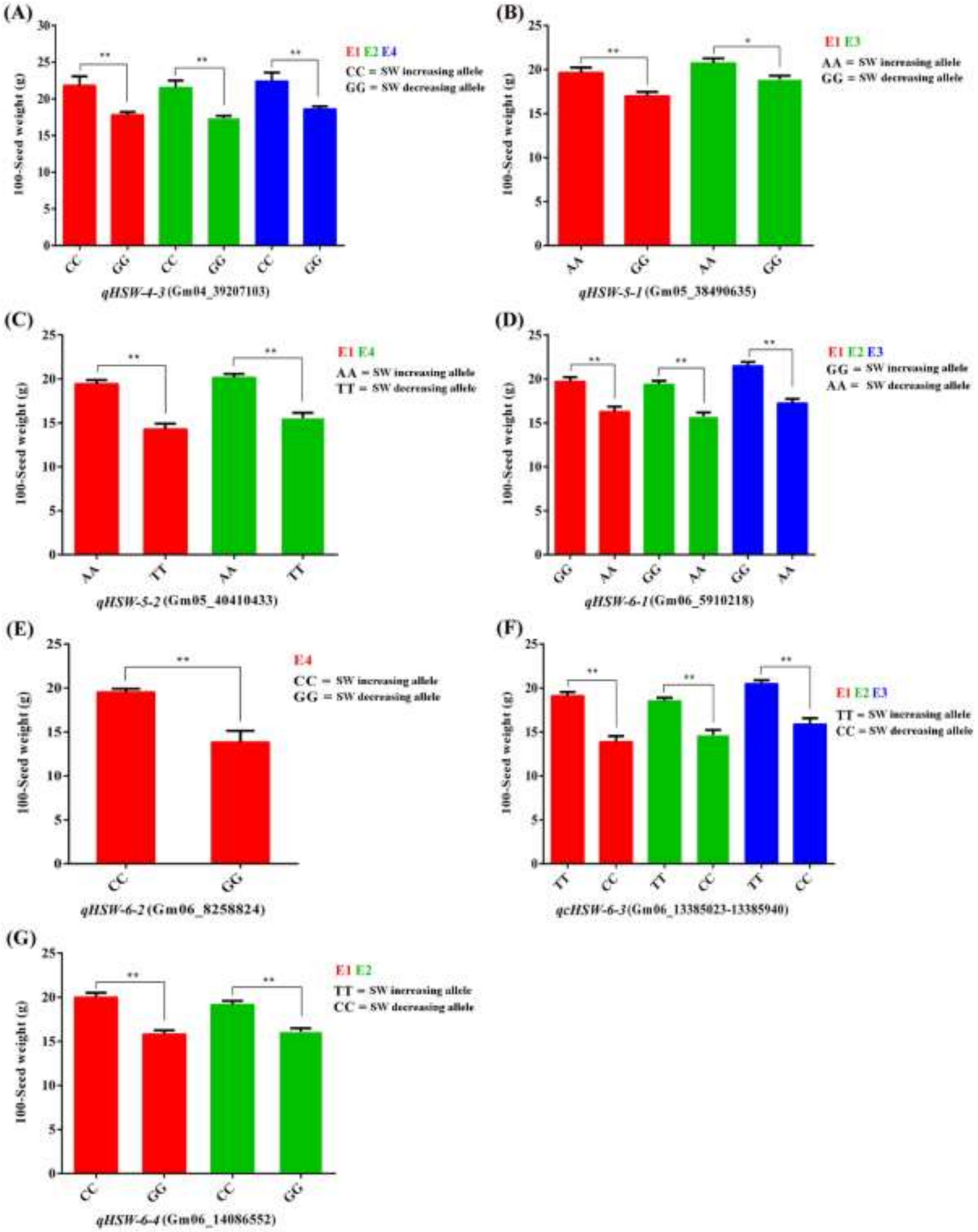

Figure S2: Phenotypic differences of 100-seed weight between accessions carrying different alleles of each QTN. These QTNs include *qHSW-4-3* (**A**), *qHSW-5-1* (**B**), *qHSW-5-2* (**C**), *qHSW-6-1* (**D**), *qHSW-6-2* (**E**), *qcHSW-6-3* (**F**), and *qHSW-6-4* (**G**). * and **: the significances at the 0.05 and 0.01 levels, respectively, using student's t-test. The error bars represent standard deviation. E1: Nanjing (2014); E2: Nanjing (2015); E3: Wuhan (2014); E4: Wuhan (2015).

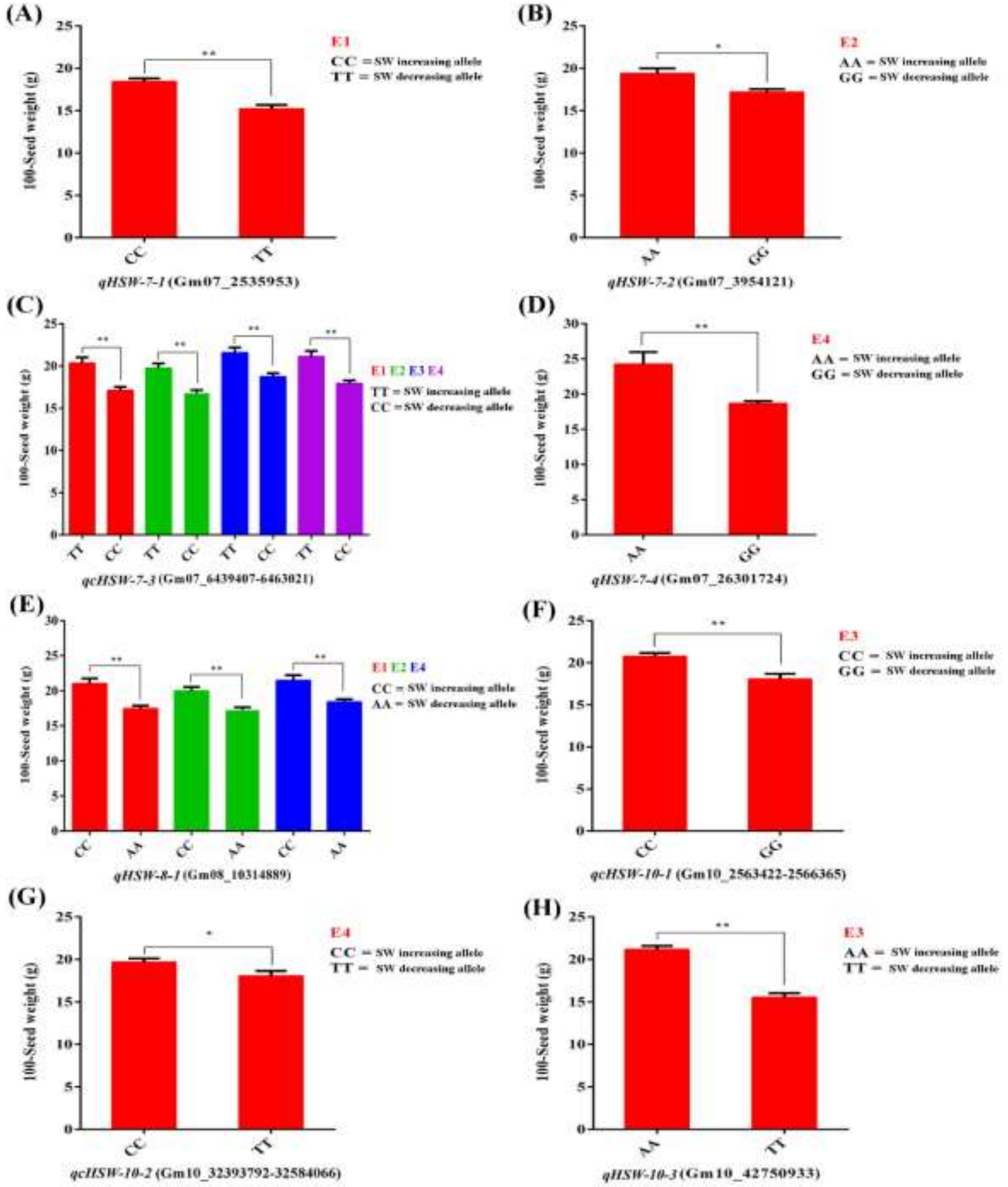

Figure S3: Phenotypic differences of 100-seed weight between accessions carrying different alleles of each QTN. These QTNs include *qHSW-7-1* (**A**), *qHSW-7-2* (**B**), *qcHSW-7-3* (**C**), *qHSW-7-4* (**D**), *qHSW-8-1* (**E**), *qcHSW-10-1* (**F**), *qcHSW-10-2* (**G**), and *qHSW-10-3* (**H**). * and **: the significances at the 0.05 and 0.01 levels, respectively, using student's t-test. The error bars represent standard deviation. E1: Nanjing (2014); E2: Nanjing (2015); E3: Wuhan (2014); E4: Wuhan (2015).

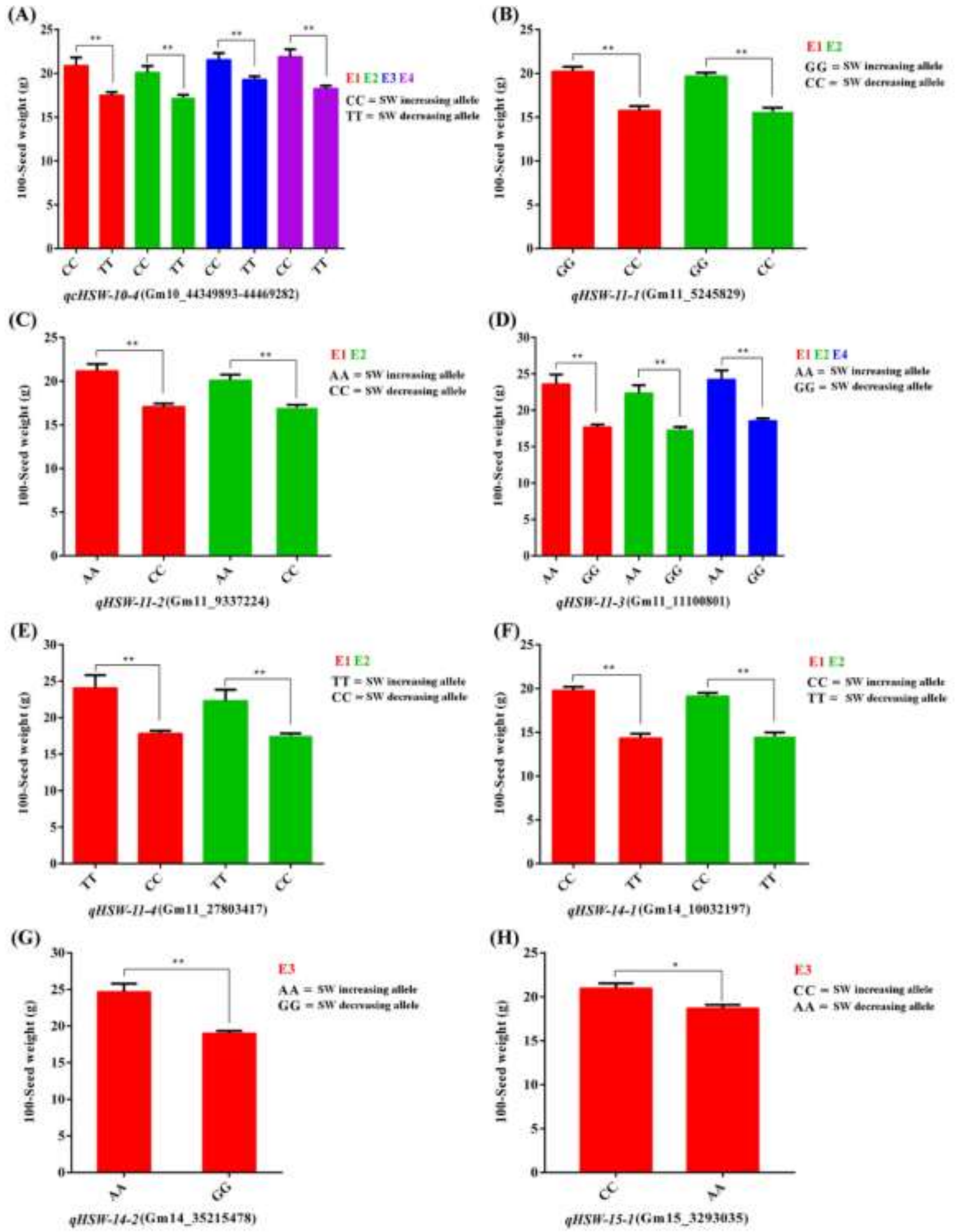

Figure S4: Phenotypic differences of 100-seed weight between accessions carrying different alleles of each QTN. These QTNs include *qcHSW-10-4* (**A**), *qHSW-11-1* (**B**), *qHSW-11-2* (**C**), *qHSW-11-3* (**D**), *qHSW-11-4* (**E**), *qHSW-14-1* (**F**), *qHSW-14-2* (**G**). * and **: the significances at the 0.05 and 0.01 levels, respectively, using student's t-test. The error bars represent standard deviation. E1: Nanjing (2014); E2: Nanjing (2015); E3: Wuhan (2014); E4: Wuhan (2015).

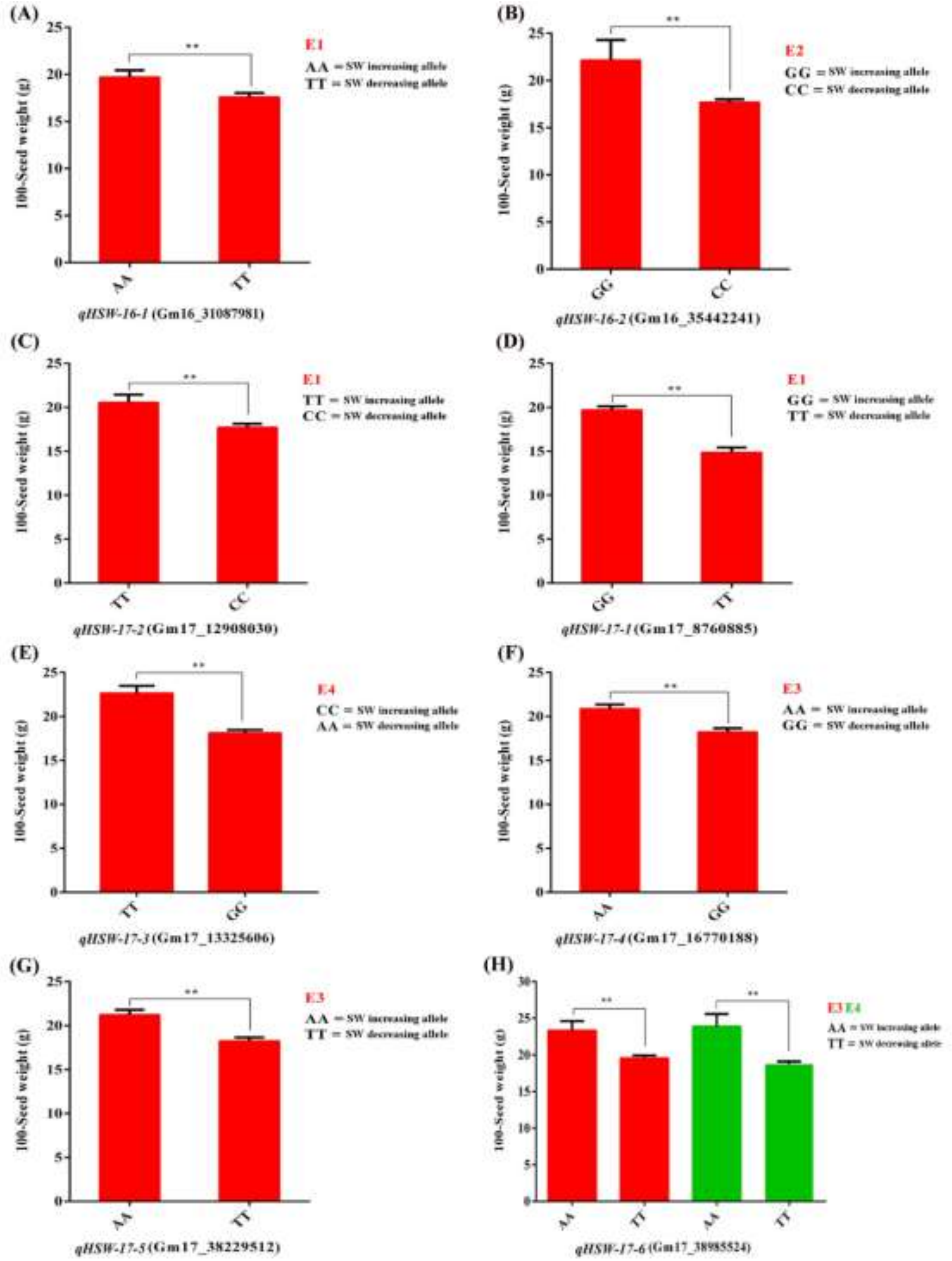

Figure S5: Phenotypic differences of 100-seed weight between accessions carrying different alleles of each QTN. These QTNs include *qHSW-16-1* (A), *qHSW-16-2* (B), *qHSW-17-2* (C), *qHSW-17-1* (D), *qHSW-17-3* (E), *qHSW-17-4* (F), *qHSW-17-5* (G), and *qHSW-17-6* (H). * and **: the significances at the 0.05 and 0.01 levels, respectively, using student's t-test. The error bars represent standard deviation. E1: Nanjing (2014); E2: Nanjing (2015); E3: Wuhan (2014); E4: Wuhan (2015).

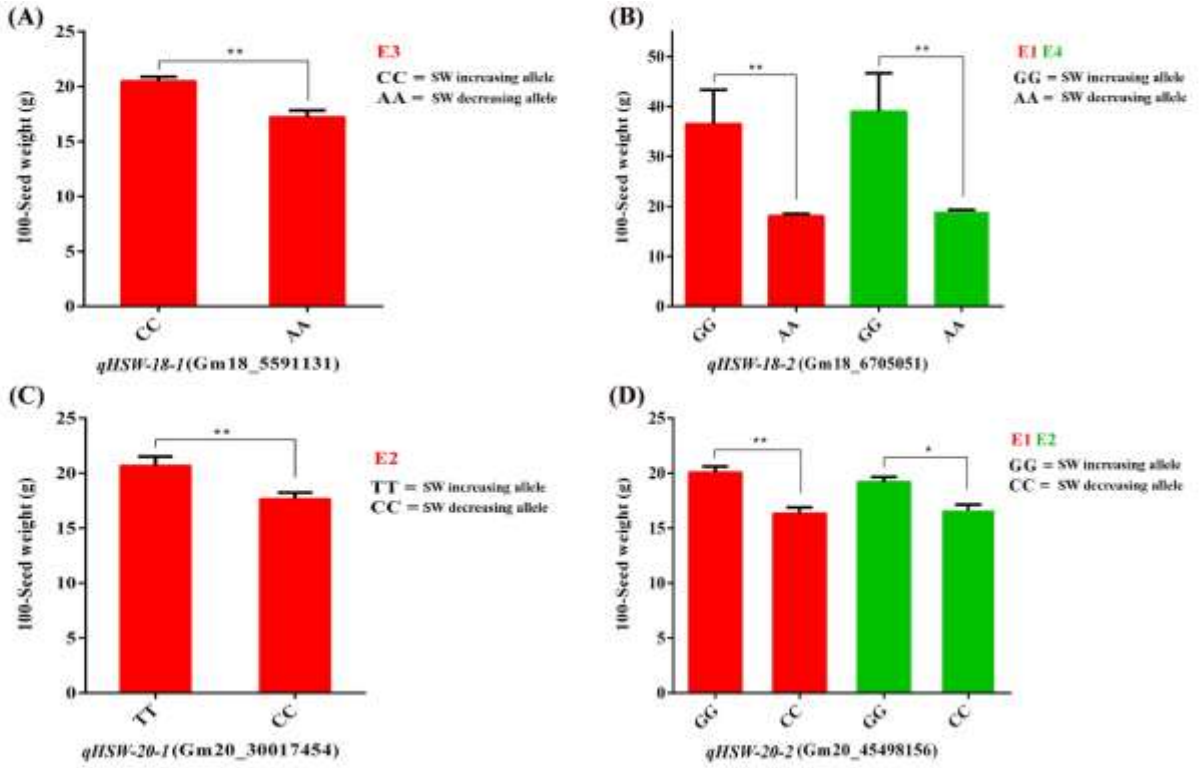

Figure S6: Phenotypic differences of 100-seed weight between accessions carrying different alleles of each QTN. These QTNs include *qHSW-18-1* (**A**), *qHSW-18-2* (**B**), *qHSW-20-1* (**C**), and *qHSW-20-2* (**D**). * and **: the significances at the 0.05 and 0.01 levels, respectively, using student's t-test. The error bars represent standard deviation. E1: Nanjing (2014); E2: Nanjing (2015); E3: Wuhan (2014); E4: Wuhan (2015).

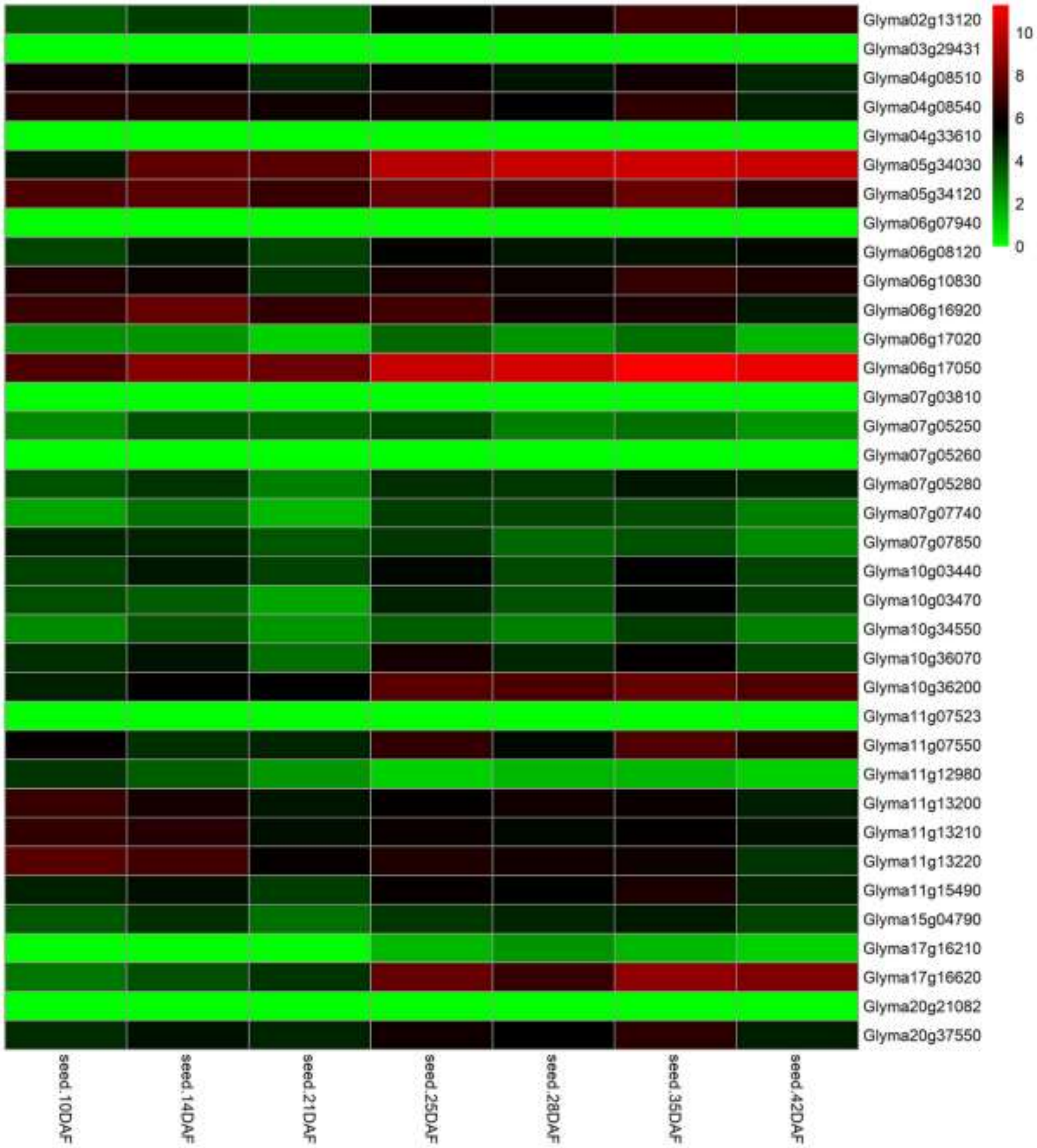

Figure S7: The expressional levels [$\log_2$(RPKM + 1)] of candidate genes associated with seed weight in seven soybean tissues

Table S1: Significant QTNs for 100-seed weight detected in four environments and BLUP model using mrMLM

Environment	SNP	Chromosome	Position (bp)	QTN effect	LOD score	r^2 (%)	MAF	Genotype code 1
E1	MM5402	2	154356	0.846	3.323	0.994	0.179	TT
E1	MM16825	4	6703334	-1.056	4.022	2.237	0.208	TT
E1	MM19336	4	37026887	1.361	5.533	4.203	0.268	AA
E1	MM16609	4	5508478	1.939	5.807	4.343	0.085	GG
E1	MM19598	4	39207103	-0.950	4.094	1.499	0.153	GG
E1	MM25032	5	38490643	1.211	7.875	4.114	0.474	AA
E1	MM27535	6	14086552	0.867	4.291	2.011	0.383	CC
E1	MM27153	6	11768194	1.030	4.094	2.101	0.214	GG
E1	MM33672	7	13593468	-3.388	6.043	2.534	0.016	CC
E1	MM31764	7	2535953	0.921	5.144	2.131	0.327	CC
E1	MM32470	7	6463021	0.816	6.264	1.828	0.423	GG
E1	MM35469	7	35642964	1.097	7.278	3.358	0.486	TT
E1	MM46239	9	37530389	0.725	3.474	1.252	0.351	AA
E1	MM50808	10	38730187	-1.058	3.837	2.132	0.167	GG
E1	MM53925	11	9337224	1.288	10.822	4.091	0.325	AA
E1	MM55590	11	27803417	-1.235	4.733	2.401	0.133	CC
E1	MM73482	15	6425141	0.893	3.581	1.778	0.262	GG
E1	MM82685	16	31087981	0.945	4.269	2.358	0.419	AA
E1	MM85374	17	12908030	1.222	6.391	3.205	0.288	TT
E1	MM105856	20	45498156	1.359	10.334	5.094	0.375	GG
E2	MM965	1	6594737	0.652	4.188	1.532	0.349	CC
E2	MM9717	2	44029773	-0.782	4.375	2.373	0.474	CC
E2	MM16825	4	6703334	-0.730	3.016	1.478	0.208	TT
E2	MM19336	4	37026887	0.921	7.579	2.659	0.268	AA
E2	MM16609	4	5508478	1.995	10.645	6.343	0.085	GG
E2	MM31195	6	49426096	0.688	4.859	1.788	0.371	TT
E2	MM33672	7	13593468	-4.669	11.728	6.644	0.016	CC
E2	MM32470	7	6463021	0.869	7.356	2.859	0.423	GG
E2	MM38232	8	10314889	0.765	3.508	1.800	0.278	CC
E2	MM53737	11	8085669	0.709	4.128	1.868	0.436	GG
E2	MM53924	11	9337184	1.037	7.669	3.641	0.325	AA
E2	MM55590	11	27803417	-1.367	5.296	4.064	0.133	CC
E2	MM83326	16	35442241	1.914	6.199	3.010	0.057	GG
E2	MM82502	16	30210113	0.709	3.290	1.949	0.468	CC
E2	MM85712	17	15319460	0.880	4.182	2.604	0.315	TT
E2	MM89037	18	6828591	0.436	3.692	0.721	0.421	AA
E2	MM95579	19	68075	0.918	4.784	2.572	0.266	AA
E2	MM103377	20	29754434	0.847	4.462	2.380	0.307	TT
E2	MM105856	20	45498156	0.807	4.224	2.480	0.375	GG
E3	MM936	1	6389301	1.146	6.128	3.866	0.333	CC
E3	MM19336	4	37026887	0.817	3.346	1.728	0.268	AA
E3	MM26278	6	5910218	0.919	3.506	2.640	0.403	GG
E3	MM27458	6	13614675	0.833	3.101	1.625	0.242	AA

E3	MM51565	10	44349907	-1.813	4.496	2.232	0.054	GG
E3	MM47906	10	2562049	-1.438	11.662	5.924	0.367	TT
E3	MM48154	10	4314357	1.121	4.686	2.035	0.143	CC
E3	MM65483	13	32343025	-0.692	3.105	1.444	0.371	CC
E3	MM73028	15	3293035	0.812	5.695	2.108	0.480	CC
E3	MM87550	17	38985524	-1.628	7.028	3.489	0.109	TT
E3	MM85898	17	16770188	-0.959	6.792	2.749	0.373	GG
E3	MM87416	17	38229512	1.217	10.371	4.749	0.468	AA
E3	MM88823	18	5591131	1.211	6.707	2.874	0.184	CC
E3	MM94676	18	58738264	1.132	5.038	3.373	0.248	TT
E3	MM103270	20	28423346	0.977	4.712	2.611	0.302	GG
E4	MM949	1	6458540	2.116	5.137	3.030	0.050	CC
E4	MM9632	2	43506875	1.178	3.733	1.710	0.129	CC
E4	MM5402	2	154356	1.013	3.923	1.621	0.179	TT
E4	MM16825	4	6703334	-1.444	4.614	4.758	0.208	TT
E4	MM32470	7	6463021	0.777	3.038	1.885	0.423	GG
E4	MM33199	7	9942944	0.966	4.415	2.792	0.371	GG
E4	MM51590	10	44469282	0.930	3.263	2.212	0.276	CC
E4	MM50106	10	32393792	-0.921	3.744	1.910	0.222	AA
E4	MM54522	11	15395305	1.044	3.722	3.392	0.361	TT
E4	MM53737	11	8085669	1.237	3.863	4.689	0.436	GG
E4	MM64405	13	26837832	1.344	4.603	4.221	0.228	AA
E4	MM76895	15	36572861	-3.778	5.874	3.582	0.018	GG
E4	MM83692	16	37165251	1.914	7.540	3.135	0.069	AA
E4	MM89014	18	6705051	-2.747	4.774	3.352	0.024	AA
E4	MM89818	18	13147331	1.394	5.498	3.920	0.196	GG
E4	MM99446	19	41142963	-1.971	3.984	2.628	0.054	CC
BLUP	MM936	1	6389301	0.700	4.219	1.760	0.333	CC
BLUP	MM16825	4	6703334	-1.129	3.172	3.564	0.208	TT
BLUP	MM26278	6	5910218	1.348	10.631	6.948	0.403	GG
BLUP	MM26594	6	8258824	1.468	3.449	3.044	0.069	CC
BLUP	MM32470	7	6463021	1.083	5.758	4.481	0.423	GG
BLUP	MM47908	10	2566365	-0.749	5.256	1.965	0.353	TT
BLUP	MM51590	10	44469282	1.282	4.570	5.153	0.276	CC
BLUP	MM50119	10	32584066	-0.921	4.757	2.510	0.276	TT
BLUP	MM53925	11	9337224	0.950	4.800	3.099	0.325	AA
BLUP	MM76895	15	36572861	-3.288	6.943	3.325	0.018	GG
BLUP	MM85419	17	13325606	1.099	4.154	3.495	0.238	TT
BLUP	MM105856	20	45498156	0.782	3.270	2.351	0.375	GG
BLUP	MM103406	20	30017454	1.108	5.781	4.371	0.325	TT

r^2%: The proportion of phenotypic variance explained by each QTN; E1: Nanjing (2014); E2: Nanjing (2015); E3: Wuhan (2014); E4: Wuhan (2015).

Table S2: Significant QTNs for 100-seed weight detected in four environments and BLUP model by using FASTmrMLM

Environment	SNP	Chromosome	Position (bp)	QTN effect	LOD score	r^2 (%)	MAF	Genotype code 1
E1	MM16609	4	5508478	1.743	6.560	3.507	0.084	GG
E1	MM19336	4	37026887	1.347	9.936	4.116	0.266	AA
E1	MM19598	4	39207103	-1.184	6.239	2.328	0.156	GG
E1	MM25031	5	38490635	1.025	8.137	2.948	0.472	AA
E1	MM27153	6	11768194	1.030	5.665	2.101	0.216	GG
E1	MM27535	6	14086552	1.163	9.213	3.615	0.384	CC
E1	MM31764	7	2535953	0.607	3.038	0.925	0.324	CC
E1	MM32470	7	6463021	0.802	5.229	1.766	0.424	GG
E1	MM35469	7	35642964	0.909	7.665	2.304	0.486	TT
E1	MM42273	8	44574454	0.836	4.853	1.626	0.310	CC
E1	MM50808	10	38730187	-1.087	6.261	2.254	0.166	GG
E1	MM51565	10	44349907	-1.834	5.341	2.003	0.054	GG
E1	MM53925	11	9337224	1.244	8.809	3.815	0.326	AA
E1	MM55590	11	27803417	-1.237	5.666	2.406	0.132	CC
E1	MM82685	16	31087981	0.851	4.950	1.915	0.420	AA
E1	MM85374	17	12908030	1.063	7.003	2.422	0.290	TT
E1	MM105856	20	45498156	1.038	7.494	2.968	0.372	GG
E2	MM936	1	6389301	0.660	5.759	1.552	0.334	CC
E2	MM13399	3	29644613	0.741	5.901	1.979	0.334	TT
E2	MM14213	3	35836201	-0.468	3.506	0.715	0.308	CC
E2	MM16609	4	5508478	1.351	6.530	2.909	0.084	GG
E2	MM16825	4	6703334	-0.739	5.169	1.514	0.206	TT
E2	MM19336	4	37026887	0.639	4.524	1.280	0.266	AA
E2	MM19598	4	39207103	-0.753	4.492	1.301	0.156	GG
E2	MM27183	6	11900234	0.567	3.624	0.737	0.176	GG
E2	MM31195	6	49426096	0.584	4.569	1.288	0.368	TT
E2	MM32470	7	6463021	0.512	3.518	0.994	0.424	GG
E2	MM35475	7	35727264	0.550	4.525	1.104	0.390	AA
E2	MM38378	8	11353439	-0.510	3.653	0.499	0.144	TT
E2	MM39297	8	17248047	0.749	7.319	1.987	0.338	AA
E2	MM48136	10	4189175	0.739	7.164	1.419	0.212	TT
E2	MM53737	11	8085669	0.755	6.340	2.123	0.436	GG
E2	MM53925	11	9337224	1.002	11.031	3.421	0.326	AA
E2	MM64714	13	28360967	0.581	4.423	0.801	0.192	TT
E2	MM68311	14	6069465	-0.908	4.548	1.274	0.092	AA
E2	MM72698	15	975135	0.717	6.348	1.581	0.262	AA
E2	MM83326	16	35442241	1.133	5.229	1.055	0.060	GG
E2	MM89037	18	6828591	0.409	3.646	0.633	0.418	AA
E2	MM103406	20	30017454	0.520	3.937	0.955	0.322	TT
E3	MM936	1	6389301	0.720	4.135	1.525	0.334	CC
E3	MM955	1	6500841	1.634	4.717	4.226	0.090	AA
E3	MM25031	5	38490635	0.623	4.297	1.244	0.472	AA
E3	MM26278	6	5910218	1.097	6.748	3.767	0.404	GG
E3	MM27416	6	13385023	0.959	4.367	1.651	0.130	TT
E3	MM47907	10	2563422	-1.366	13.802	4.914	0.306	GG
E3	MM48154	10	4314357	1.098	5.003	1.954	0.142	CC

E3	MM51364	10	42750933	0.986	3.966	2.540	0.238	AA
E3	MM64714	13	28360967	0.645	3.493	0.816	0.192	TT
E3	MM73028	15	3293035	0.661	3.680	1.398	0.480	CC
E3	MM85898	17	16770188	-1.433	13.316	6.135	0.374	GG
E3	MM87411	17	38202340	1.176	9.428	4.328	0.408	GG
E3	MM87550	17	38985524	-1.792	9.497	4.232	0.108	TT
E3	MM88823	18	5591131	1.172	6.170	2.694	0.182	CC
E4	MM944	1	6435399	2.319	7.858	7.890	0.084	CC
E4	MM7017	2	11278769	1.023	5.656	3.103	0.380	CC
E4	MM9632	2	43506875	1.044	3.146	1.344	0.132	CC
E4	MM15918	4	640399	-1.673	4.066	2.145	0.056	GG
E4	MM21642	5	3322804	1.018	5.280	3.207	0.406	TT
E4	MM32470	7	6463021	0.864	4.767	2.330	0.424	GG
E4	MM35063	7	30137532	-1.855	6.919	4.653	0.110	TT
E4	MM50106	10	32393792	-1.049	4.960	2.479	0.228	AA
E4	MM51590	10	44469282	1.008	3.640	2.598	0.278	CC
E4	MM54798	11	17338166	-2.606	4.914	3.656	0.040	CC
E4	MM85154	17	11605751	-1.466	3.013	2.118	0.058	TT
E4	MM85419	17	13325606	1.097	3.716	2.843	0.244	TT
BLUP	MM7017	2	11278769	0.527	5.226	1.008	0.380	CC
BLUP	MM11062	3	1445425	-0.717	4.230	0.826	0.100	CC
BLUP	MM12414	3	17127049	0.764	3.869	0.612	0.108	GG
BLUP	MM14469	3	37435877	-1.358	4.030	0.677	0.024	TT
BLUP	MM16825	4	6703334	-0.927	7.665	2.400	0.206	TT
BLUP	MM19336	4	37026887	0.593	4.561	1.114	0.266	AA
BLUP	MM25029	5	38490610	0.630	6.510	1.483	0.386	GG
BLUP	MM26278	6	5910218	0.984	11.684	3.699	0.404	GG
BLUP	MM26594	6	8258824	1.179	5.492	1.963	0.068	CC
BLUP	MM27535	6	14086552	0.787	8.741	2.308	0.384	CC
BLUP	MM32470	7	6463021	0.795	8.641	2.414	0.424	GG
BLUP	MM35475	7	35727264	0.646	6.327	1.534	0.390	AA
BLUP	MM39297	8	17248047	0.860	10.368	2.646	0.338	AA
BLUP	MM40750	8	34373746	0.863	5.270	1.089	0.108	AA
BLUP	MM47908	10	2566365	-0.651	6.330	1.484	0.354	TT
BLUP	MM48390	10	6256342	-0.425	4.176	0.693	0.424	GG
BLUP	MM50119	10	32584066	-0.653	4.959	1.260	0.282	TT
BLUP	MM51467	10	43493868	0.417	4.425	0.644	0.376	CC
BLUP	MM51590	10	44469282	0.661	5.421	1.372	0.278	CC
BLUP	MM53925	11	9337224	0.552	3.099	1.045	0.326	AA
BLUP	MM62089	13	5475253	-0.972	3.834	0.940	0.052	CC
BLUP	MM85419	17	13325606	0.729	4.969	1.538	0.244	TT
BLUP	MM100272	19	47086601	-0.667	7.340	1.499	0.308	GG
BLUP	MM103298	20	29003225	0.592	5.847	1.195	0.310	CC

r^2%: The proportion of phenotypic variance explained by each QTN; E1: Nanjing (2014); E2: Nanjing (2015); E3: Wuhan (2014); E4: Wuhan (2015).

Table S3: Significant QTNs for 100-seed weight detected in four environments and BLUP model by using FASTmrEMMA

Environment	SNP	Chromosome	Position (bp)	QTN effect	LOD score	r^2 (%)	MAF	Genotype code 1
E1	MM965	1	6594737	1.770	4.462	1.945	0.350	CC
E1	MM16825	4	6703334	-2.317	4.581	2.275	0.206	TT
E1	MM26278	6	5910218	1.924	3.947	2.425	0.404	GG
E1	MM27419	6	13385940	3.445	6.906	3.591	0.136	TT
E1	MM27535	6	14086552	2.278	6.168	3.426	0.384	CC
E1	MM32461	7	6439407	2.253	6.545	3.414	0.416	TT
E1	MM53925	11	9337224	2.950	7.561	5.359	0.326	AA
E1	MM62348	13	6971521	1.958	3.221	1.339	0.148	TT
E1	MM84793	17	8760885	1.810	3.856	1.701	0.252	GG
E1	MM105856	20	45498156	1.827	4.207	1.933	0.372	GG
E2	MM965	1	6594737	1.610	3.884	2.223	0.350	CC
E2	MM16825	4	6703334	-3.012	8.642	5.311	0.206	TT
E2	MM19336	4	37026887	1.716	4.118	2.154	0.266	AA
E2	MM26278	6	5910218	1.959	4.567	3.474	0.404	GG
E2	MM27535	6	14086552	2.373	9.438	5.136	0.384	CC
E2	MM31998	7	3954121	1.365	4.010	1.667	0.362	AA
E2	MM32461	7	6439407	1.787	4.562	2.967	0.416	TT
E2	MM51590	10	44469282	1.725	3.807	2.309	0.278	CC
E2	MM53925	11	9337224	1.442	3.319	1.768	0.326	AA
E2	MM70626	14	30115761	2.625	7.031	4.107	0.216	CC
E3	MM936	1	6389301	2.302	5.789	3.599	0.334	CC
E3	MM955	1	6500841	4.079	6.304	2.933	0.090	AA
E3	MM26278	6	5910218	2.719	8.486	5.524	0.404	GG
E3	MM47907	10	2563422	-3.230	12.963	6.812	0.306	GG
E3	MM64712	13	28354636	1.684	3.719	1.382	0.198	TT
E3	MM70868	14	35215478	2.369	4.530	2.120	0.156	AA
E3	MM85898	17	16770188	-2.294	5.337	3.905	0.374	GG
E3	MM87416	17	38229512	3.078	9.775	7.263	0.464	AA
E3	MM87550	17	38985524	-3.384	5.923	3.396	0.108	TT
E4	MM7017	2	11278769	1.978	4.446	2.851	0.380	CC
E4	MM9632	2	43506875	2.777	4.114	2.531	0.132	CC
E4	MM16825	4	6703334	-3.331	7.091	5.347	0.206	TT
E4	MM21642	5	3322804	1.635	3.182	2.054	0.406	TT
E4	MM32470	7	6463021	2.059	4.538	3.286	0.424	GG
E4	MM34903	7	26301724	-3.488	5.394	3.406	0.112	GG
E4	MM50713	10	38186126	-2.147	4.314	2.863	0.270	TT
E4	MM51590	10	44469282	2.347	4.558	3.521	0.278	CC
BLUP	MM893	1	5813393	1.710	5.821	2.633	0.364	TT
BLUP	MM16825	4	6703334	-2.585	7.605	3.947	0.206	TT
BLUP	MM25031	5	38490635	1.247	3.291	1.508	0.472	AA
BLUP	MM26278	6	5910218	1.735	4.588	2.748	0.404	GG
BLUP	MM27416	6	13385023	2.134	4.008	1.681	0.130	TT
BLUP	MM27535	6	14086552	1.770	6.301	2.881	0.384	CC
BLUP	MM32461	7	6439407	1.706	5.556	2.729	0.416	TT

BLUP	MM47908	10	2566365	-1.140	3.563	1.119	0.354	TT
BLUP	MM51590	10	44469282	1.444	3.389	1.634	0.278	CC
BLUP	MM53750	11	8139676	1.038	3.253	1.024	0.440	AA
BLUP	MM53925	11	9337224	1.502	3.218	1.936	0.326	AA
BLUP	MM82492	16	30145369	2.759	3.435	1.171	0.042	AA
BLUP	MM87550	17	38985524	-2.272	4.364	1.870	0.108	TT

r^2%: The proportion of phenotypic variance explained by each QTN; E1: Nanjing (2014); E2: Nanjing (2015); E3: Wuhan (2014); E4: Wuhan (2015).

Table S4: Significant QTNs for 100-seed weight detected in four environments and BLUP model by using pLARmEB

Environment	SNP	Chromosome	Position (bp)	QTN effect	LOD score	r^2 (%)	MAF	Genotype code 1
E1	MM37	1	350241	0.866	6.281	1.541	0.226	CC
E1	MM690	1	4558041	2.547	8.459	5.622	0.052	AA
E1	MM2359	1	28459059	-0.707	3.343	0.628	0.120	AA
E1	MM5938	2	3711238	-1.156	5.948	1.589	0.118	TT
E1	MM12414	3	17127049	1.494	6.095	1.679	0.108	GG
E1	MM19598	4	39207103	-0.859	4.840	1.227	0.156	GG
E1	MM25597	6	1053761	0.603	4.809	1.013	0.464	AA
E1	MM27419	6	13385940	0.813	3.132	0.980	0.136	TT
E1	MM27535	6	14086552	1.177	9.285	3.704	0.384	CC
E1	MM31764	7	2535953	1.129	10.456	3.204	0.324	CC
E1	MM32848	7	8157652	0.833	4.777	1.881	0.366	GG
E1	MM34535	7	19338710	-2.595	4.754	1.486	0.020	GG
E1	MM39334	8	17444010	0.679	3.567	0.967	0.246	TT
E1	MM53925	11	9337224	0.844	4.004	1.758	0.326	AA
E1	MM56592	11	36968317	0.727	4.909	1.097	0.246	CC
E1	MM57453	12	3529776	-0.766	4.419	0.795	0.128	AA
E1	MM61073	12	38071047	1.235	5.677	1.653	0.098	GG
E1	MM68891	14	10032197	0.798	3.772	1.364	0.252	CC
E1	MM82685	16	31087981	0.642	3.586	1.090	0.420	AA
E1	MM84793	17	8760885	0.811	4.392	1.425	0.252	GG
E1	MM99152	19	39191055	0.576	3.836	0.913	0.398	CC
E2	MM916	1	6178120	1.515	5.436	3.211	0.072	GG
E2	MM12414	3	17127049	1.037	4.013	1.118	0.108	GG
E2	MM14213	3	35836201	-0.864	7.181	2.438	0.308	CC
E2	MM16825	4	6703334	-0.982	5.820	2.674	0.206	TT
E2	MM27377	6	13121226	-0.728	4.268	1.152	0.166	CC
E2	MM45169	9	27484544	-0.745	4.005	1.443	0.172	GG
E2	MM48137	10	4189178	0.904	5.434	1.602	0.148	CC
E2	MM51564	10	44349893	-1.809	6.078	2.871	0.056	GG
E2	MM53232	11	4695722	1.666	4.285	1.658	0.032	AA
E2	MM66299	13	37459905	0.814	6.171	2.241	0.322	TT
E2	MM68891	14	10032197	1.526	15.344	6.901	0.252	CC
E2	MM72310	14	48268269	-0.497	3.724	0.941	0.458	CC
E2	MM73894	15	9153526	-0.491	3.189	0.922	0.436	GG
E2	MM74450	15	12641182	-1.707	4.562	2.714	0.040	TT
E2	MM85721	17	15374064	0.788	3.796	1.399	0.172	CC
E2	MM87010	17	35153865	0.615	3.719	1.462	0.498	AA
E2	MM100272	19	47086601	-0.566	3.706	1.070	0.308	GG
E2	MM103406	20	30017454	0.579	3.518	1.181	0.322	TT
E3	MM6124	2	5269353	1.030	5.304	2.182	0.178	AA
E3	MM9632	2	43506875	0.964	4.065	1.150	0.132	CC
E3	MM24828	5	36813389	0.808	4.505	1.529	0.216	GG
E3	MM26256	6	5684370	0.994	5.278	2.881	0.312	TT
E3	MM31319	6	50185454	1.099	4.307	2.043	0.110	AA

E3	MM32470	7	6463021	0.897	6.864	2.518	0.424	GG
E3	MM47908	10	2566365	-0.787	5.199	1.774	0.354	TT
E3	MM51564	10	44349893	-2.208	6.347	3.533	0.056	GG
E3	MM53105	11	3687730	-0.726	3.862	1.682	0.468	GG
E3	MM54638	11	16203718	1.691	7.912	3.878	0.092	GG
E3	MM60292	12	32790779	1.094	3.969	1.235	0.084	GG
E3	MM73028	15	3293035	1.076	9.037	3.703	0.480	CC
E3	MM73367	15	5667402	-0.992	7.585	3.150	0.478	GG
E3	MM85898	17	16770188	-0.932	4.816	2.596	0.374	GG
E3	MM87415	17	38211926	1.012	7.273	3.176	0.388	TT
E3	MM105864	20	45603666	0.732	4.378	1.311	0.256	GG
E4	MM944	1	6435399	1.767	5.782	2.738	0.084	CC
E4	MM7017	2	11278769	0.794	4.144	1.115	0.380	CC
E4	MM16825	4	6703334	-1.182	5.745	1.906	0.206	TT
E4	MM21637	5	3302544	0.846	5.611	1.366	0.472	GG
E4	MM24731	5	36060949	0.840	4.918	1.217	0.322	GG
E4	MM26594	6	8258824	1.476	4.701	1.501	0.068	CC
E4	MM32470	7	6463021	1.034	7.490	1.993	0.424	GG
E4	MM42613	9	95010	0.660	4.373	0.802	0.392	GG
E4	MM43737	9	7248257	-0.648	4.499	0.718	0.370	CC
E4	MM50713	10	38186126	-0.781	3.594	0.933	0.270	TT
E4	MM50951	10	39768247	0.642	4.035	0.642	0.362	CC
E4	MM51590	10	44469282	1.128	6.636	1.944	0.278	CC
E4	MM53455	11	6080251	1.095	5.456	1.208	0.150	AA
E4	MM71997	14	46610530	0.506	3.419	0.388	0.264	GG
E4	MM85154	17	11605751	-1.181	3.116	0.822	0.058	TT
E4	MM87418	17	38233161	0.564	3.161	0.609	0.466	TT
E4	MM87550	17	38985524	-1.669	8.053	2.184	0.108	TT
E4	MM89014	18	6705051	-3.984	8.392	4.214	0.024	AA
BLUP	MM5515	2	977677	-1.249	3.476	0.914	0.038	CC
BLUP	MM7017	2	11278769	0.745	5.860	1.650	0.380	CC
BLUP	MM16825	4	6703334	-0.797	3.998	1.453	0.206	TT
BLUP	MM20963	4	48154457	0.513	3.962	0.699	0.282	CC
BLUP	MM25621	6	1262823	-0.936	3.515	0.554	0.048	AA
BLUP	MM26278	6	5910218	0.719	4.575	1.616	0.404	GG
BLUP	MM27535	6	14086552	0.457	3.038	0.635	0.384	CC
BLUP	MM31764	7	2535953	0.764	5.383	1.668	0.324	CC
BLUP	MM32470	7	6463021	0.749	6.296	1.751	0.424	GG
BLUP	MM42647	9	336033	1.885	9.519	4.674	0.066	CC
BLUP	MM51355	10	42618806	0.957	6.192	1.704	0.166	GG
BLUP	MM54178	11	11136355	-1.425	9.071	3.710	0.146	AA
BLUP	MM61802	13	3246710	-1.912	5.159	1.098	0.022	CC
BLUP	MM68891	14	10032197	1.053	9.889	2.710	0.252	CC
BLUP	MM70868	14	35215478	0.968	6.165	1.340	0.156	AA
BLUP	MM72041	14	46857740	0.423	4.250	0.555	0.450	GG
BLUP	MM87550	17	38985524	-1.167	6.255	1.790	0.108	TT

BLUP	MM100873	20	669328	-0.472	3.537	0.684	0.394	GG
BLUP	MM103406	20	30017454	0.893	8.385	2.322	0.322	TT
BLUP	MM105106	20	40753657	-0.690	3.541	0.837	0.126	CC

r^2%: The proportion of phenotypic variance explained by each QTN; E1: Nanjing (2014); E2: Nanjing (2015); E3: Wuhan (2014); E4: Wuhan (2015).

Table S5: Significant QTNs for 100-seed weight detected in four environments and BLUP model by using pKWmEB

Environment	SNP	Chromosome	Position (bp)	QTN effect	LOD score	r^2 (%)	MAF	Genotype code 1
E1	MM14469	3	37435877	-2.206	3.423	2.570	0.024	TT
E1	MM16825	4	6703334	-0.783	3.505	1.592	0.208	TT
E1	MM16694	4	5939386	1.000	3.525	1.796	0.065	CC
E1	MM26278	6	5910218	1.027	7.139	3.701	0.403	GG
E1	MM27535	6	14086552	0.745	4.378	1.486	0.383	CC
E1	MM27419	6	13385940	1.167	4.824	2.993	0.137	TT
E1	MM31195	6	49426096	0.608	3.737	1.970	0.371	TT
E1	MM32470	7	6463021	1.271	10.231	4.065	0.423	GG
E1	MM42791	9	1200568	-0.896	3.471	2.575	0.135	TT
E1	MM43097	9	3154319	-0.674	3.805	1.711	0.202	AA
E1	MM53925	11	9337224	1.182	6.573	5.502	0.325	AA
E1	MM54162	11	11100801	-1.348	4.688	2.697	0.121	GG
E1	MM55590	11	27803417	-0.955	4.445	3.629	0.133	CC
E1	MM62348	13	6971521	1.020	5.050	2.931	0.145	TT
E1	MM73482	15	6425141	0.908	5.450	4.195	0.262	GG
E1	MM84793	17	8760885	0.663	3.614	2.027	0.254	GG
E1	MM105856	20	45498156	0.761	5.259	2.827	0.375	GG
E2	MM965	1	6594737	0.669	5.534	2.691	0.349	CC
E2	MM9716	2	44022011	0.598	6.193	1.703	0.315	CC
E2	MM13399	3	29644613	0.591	4.609	1.595	0.337	TT
E2	MM19437	4	37957928	0.821	5.258	1.359	0.202	GG
E2	MM16825	4	6703334	-0.976	8.241	2.587	0.208	TT
E2	MM16318	4	3552232	0.383	3.200	1.163	0.286	TT
E2	MM27416	6	13385023	0.748	3.879	2.031	0.131	TT
E2	MM34637	7	20198419	-1.063	3.061	3.609	0.046	CC
E2	MM32470	7	6463021	0.767	7.805	2.235	0.423	GG
E2	MM31998	7	3954121	0.511	5.627	1.947	0.361	AA
E2	MM38370	8	11330945	-0.673	4.407	1.090	0.133	TT
E2	MM45169	9	27484544	-0.499	3.434	1.747	0.173	GG
E2	MM43097	9	3154319	-0.544	3.794	1.163	0.202	AA
E2	MM48137	10	4189178	0.799	6.154	1.628	0.145	CC
E2	MM54162	11	11100801	-0.930	5.252	2.143	0.121	GG
E2	MM53925	11	9337224	0.836	7.827	3.043	0.325	AA
E2	MM55590	11	27803417	-0.572	3.685	1.531	0.133	CC
E2	MM66299	13	37459905	0.601	6.239	1.526	0.321	TT
E2	MM68633	14	8383046	0.824	6.260	2.290	0.153	AA
E2	MM70646	14	30489527	0.620	3.794	1.854	0.212	AA
E2	MM78588	15	50273729	-0.365	3.493	1.854	0.407	GG
E2	MM78045	15	47621157	0.660	7.269	3.869	0.389	TT
E2	MM85497	17	13842333	-0.942	11.040	2.562	0.351	TT
E2	MM89162	18	7542698	-0.741	6.158	2.252	0.216	CC
E2	MM103406	20	30017454	0.650	6.236	3.530	0.325	TT
E3	MM955	1	6500841	1.360	5.000	4.950	0.091	AA
E3	MM936	1	6389301	0.584	4.488	3.041	0.333	CC

E3	MM164	1	1419778	-0.852	5.936	1.049	0.173	CC
E3	MM545	1	3653563	-0.476	3.644	2.131	0.208	GG
E3	MM16825	4	6703334	-0.485	3.040	1.059	0.208	TT
E3	MM16721	4	6135792	-0.825	4.308	1.632	0.161	CC
E3	MM26278	6	5910218	0.900	7.166	3.971	0.403	GG
E3	MM41477	8	40899042	0.737	3.679	3.507	0.079	TT
E3	MM43058	9	2816883	1.883	6.887	3.049	0.054	GG
E3	MM51364	10	42750933	1.400	10.756	5.472	0.240	AA
E3	MM47907	10	2563422	-1.213	12.781	5.523	0.304	GG
E3	MM73028	15	3293035	0.637	4.825	1.930	0.480	CC
E3	MM85898	17	16770188	-0.831	6.036	4.093	0.373	GG
E3	MM87416	17	38229512	1.112	10.718	5.312	0.468	AA
E3	MM87550	17	38985524	-1.346	7.872	4.605	0.109	TT
E3	MM88823	18	5591131	1.514	12.841	4.352	0.184	CC
E3	MM89008	18	6676039	0.627	3.649	1.537	0.196	TT
E4	MM529	1	3581748	0.949	8.386	3.447	0.454	CC
E4	MM9632	2	43506875	1.118	4.566	1.880	0.129	CC
E4	MM7017	2	11278769	0.747	4.615	1.473	0.379	CC
E4	MM14469	3	37435877	-2.551	3.187	3.244	0.024	TT
E4	MM19598	4	39207103	-1.193	5.905	4.753	0.153	GG
E4	MM25151	5	39427857	0.848	3.869	1.994	0.204	AA
E4	MM34903	7	26301724	-1.394	5.100	3.496	0.113	GG
E4	MM35498	7	35840564	-0.653	4.247	2.145	0.331	TT
E4	MM32470	7	6463021	0.963	7.120	3.601	0.423	GG
E4	MM38232	8	10314889	0.696	4.202	1.777	0.278	CC
E4	MM48390	10	6256342	-0.655	4.695	2.145	0.423	GG
E4	MM50106	10	32393792	-0.558	3.448	1.821	0.222	AA
E4	MM54162	11	11100801	-1.393	4.522	2.883	0.121	GG
E4	MM56677	11	37424359	1.231	3.542	3.491	0.071	GG
E4	MM54798	11	17338166	-2.221	5.612	3.336	0.040	CC
E4	MM64405	13	26837832	0.835	4.089	3.423	0.228	AA
E4	MM71225	14	41609545	0.961	3.986	3.326	0.161	CC
E4	MM75176	15	17148291	-1.909	8.683	5.377	0.089	AA
BLUP	MM13399	3	29644613	0.549	3.674	1.493	0.337	TT
BLUP	MM14469	3	37435877	-1.684	4.983	1.258	0.024	TT
BLUP	MM11686	3	5450866	-0.410	3.650	1.375	0.270	GG
BLUP	MM16825	4	6703334	-0.674	3.750	1.121	0.208	TT
BLUP	MM27535	6	14086552	0.634	5.828	2.544	0.383	CC
BLUP	MM31195	6	49426096	0.810	10.065	3.706	0.371	TT
BLUP	MM32461	7	6439407	0.999	12.387	4.228	0.415	TT
BLUP	MM34903	7	26301724	-0.744	4.170	1.856	0.113	GG
BLUP	MM34030	7	15953694	-0.946	4.695	3.009	0.101	TT
BLUP	MM35475	7	35727264	0.536	4.997	2.263	0.389	AA
BLUP	MM39540	8	18616365	0.436	4.233	1.425	0.377	GG
BLUP	MM51564	10	44349893	-1.081	3.091	1.915	0.057	GG
BLUP	MM51590	10	44469282	0.664	4.695	2.317	0.276	CC

BLUP	MM50119	10	32584066	-0.624	4.948	1.475	0.276	TT
BLUP	MM54162	11	11100801	-0.760	3.199	1.346	0.121	GG
BLUP	MM53329	11	5245829	0.501	3.386	2.028	0.399	GG
BLUP	MM64859	13	29040876	-0.474	3.747	1.640	0.218	TT
BLUP	MM70868	14	35215478	0.664	3.519	1.472	0.149	AA
BLUP	MM85419	17	13325606	0.983	8.589	4.984	0.238	TT
BLUP	MM100832	20	358174	0.563	4.004	4.024	0.470	CC

r^2%: The proportion of phenotypic variance explained by each QTN; E1: Nanjing (2014); E2: Nanjing (2015); E3: Wuhan (2014); E4: Wuhan (2015).

Table S6: Significant QTNs for 100-seed weight detected in four environments and BLUP model by using ISIS EM-BLASSO

Environment	SNP	Chromosome	Position (bp)	QTN effect	LOD score	r^2 (%)	MAF	Genotype code 1
E1	MM9339	2	41462912	0.753	5.621	1.371	0.290	AA
E1	MM9632	2	43506875	1.028	4.801	1.146	0.132	CC
E1	MM13399	3	29644613	0.783	4.718	1.597	0.334	TT
E1	MM15871	4	348778	-1.927	6.075	1.758	0.040	CC
E1	MM20963	4	48154457	0.587	3.225	0.803	0.282	CC
E1	MM25244	5	40055670	-0.728	3.642	0.667	0.126	GG
E1	MM25289	5	40410433	0.918	5.244	1.752	0.222	AA
E1	MM26278	6	5910218	0.948	7.087	2.467	0.404	GG
E1	MM27535	6	14086552	0.694	4.921	1.286	0.384	CC
E1	MM31764	7	2535953	0.660	4.576	1.095	0.324	CC
E1	MM32461	7	6439407	0.945	8.393	2.428	0.416	TT
E1	MM38232	8	10314889	0.685	4.338	1.046	0.280	CC
E1	MM42663	9	445781	1.031	8.642	2.808	0.378	CC
E1	MM48398	10	6377706	-0.540	4.626	0.818	0.472	GG
E1	MM51565	10	44349907	-1.194	3.119	0.848	0.054	GG
E1	MM53329	11	5245829	0.662	4.520	1.207	0.396	GG
E1	MM60323	12	33108268	1.009	8.945	2.778	0.406	TT
E1	MM71225	14	41609545	0.970	4.972	1.592	0.160	CC
E1	MM82502	16	30210113	1.053	8.070	3.108	0.468	CC
E1	MM85374	17	12908030	0.734	4.147	1.157	0.290	TT
E1	MM89014	18	6705051	-2.784	5.847	3.028	0.024	AA
E1	MM103489	20	30581992	1.487	5.057	0.956	0.040	TT
E2	MM4364	1	48290180	0.760	5.069	1.156	0.142	GG
E2	MM13399	3	29644613	0.824	6.650	2.442	0.334	TT
E2	MM16825	4	6703334	-1.098	7.755	3.341	0.206	TT
E2	MM25597	6	1053761	0.598	5.343	1.373	0.464	AA
E2	MM26278	6	5910218	0.893	8.110	3.023	0.404	GG
E2	MM27535	6	14086552	0.749	7.141	2.070	0.384	CC
E2	MM31998	7	3954121	0.492	3.563	0.865	0.362	AA
E2	MM32461	7	6439407	1.255	16.609	5.914	0.416	TT
E2	MM35437	7	35426786	-0.533	4.991	1.007	0.352	GG
E2	MM38370	8	11330945	-0.983	6.773	1.853	0.132	TT
E2	MM47931	10	2734905	0.608	3.967	0.961	0.188	CC
E2	MM51590	10	44469282	1.125	10.738	3.933	0.278	CC
E2	MM53329	11	5245829	0.773	7.349	2.270	0.396	GG
E2	MM64908	13	29309070	-1.475	4.243	2.374	0.044	GG
E2	MM70618	14	30028387	0.833	4.956	1.725	0.198	AA
E2	MM83326	16	35442241	1.070	4.641	0.940	0.060	GG
E2	MM85497	17	13842333	-0.964	9.047	3.315	0.352	TT
E2	MM103458	20	30376518	1.179	7.042	1.877	0.082	CC
E3	MM955	1	6500841	1.715	6.150	4.657	0.090	AA
E3	MM16721	4	6135792	-0.998	4.757	1.984	0.164	CC
E3	MM26278	6	5910218	1.039	7.465	3.378	0.404	GG
E3	MM43058	9	2816883	1.737	4.065	2.323	0.054	GG
E3	MM47908	10	2566365	-1.075	9.046	3.309	0.354	TT
E3	MM51364	10	42750933	1.502	9.420	5.893	0.238	AA

E3	MM73028	15	3293035	0.843	5.780	2.273	0.480	CC
E3	MM85898	17	16770188	-1.299	10.137	5.044	0.374	GG
E3	MM87415	17	38211926	0.968	6.207	2.905	0.388	TT
E3	MM87550	17	38985524	-1.692	8.717	3.772	0.108	TT
E3	MM88823	18	5591131	1.492	10.008	4.363	0.182	CC
E4	MM5515	2	977677	-2.061	3.308	2.484	0.038	CC
E4	MM9632	2	43506875	1.166	4.736	1.678	0.132	CC
E4	MM15918	4	640399	-1.129	3.337	0.977	0.056	GG
E4	MM16825	4	6703334	-1.327	7.231	4.018	0.206	TT
E4	MM24851	5	36992018	1.119	5.727	1.740	0.120	AA
E4	MM25289	5	40410433	1.204	6.888	3.423	0.222	AA
E4	MM25798	6	2683134	0.929	8.165	2.763	0.496	GG
E4	MM26594	6	8258824	1.525	4.448	2.678	0.068	CC
E4	MM27071	6	11374117	0.662	3.492	1.067	0.300	TT
E4	MM32470	7	6463021	0.950	6.768	2.814	0.424	GG
E4	MM34903	7	26301724	-1.264	3.953	2.404	0.112	GG
E4	MM42647	9	336033	1.343	3.232	2.367	0.066	CC
E4	MM50119	10	32584066	-0.901	5.367	1.957	0.282	TT
E4	MM51590	10	44469282	1.519	10.077	5.904	0.278	CC
E4	MM76895	15	36572861	-4.614	7.812	5.342	0.018	GG
E4	MM103497	20	30608644	1.109	3.632	1.662	0.084	GG
BLUP	MM935	1	6370772	1.034	3.737	0.887	0.040	CC
BLUP	MM5515	2	977677	-1.024	3.438	0.752	0.038	CC
BLUP	MM9632	2	43506875	0.838	5.666	1.061	0.132	CC
BLUP	MM16825	4	6703334	-0.798	5.441	1.781	0.206	TT
BLUP	MM25289	5	40410433	0.685	5.585	1.356	0.222	AA
BLUP	MM26278	6	5910218	0.917	10.810	3.217	0.404	GG
BLUP	MM26594	6	8258824	1.061	4.403	1.589	0.068	CC
BLUP	MM27416	6	13385023	0.713	3.642	1.116	0.130	TT
BLUP	MM31998	7	3954121	0.556	5.177	1.119	0.362	AA
BLUP	MM32461	7	6439407	1.131	18.089	4.845	0.416	TT
BLUP	MM38232	8	10314889	0.469	3.174	0.682	0.280	CC
BLUP	MM42791	9	1200568	-0.738	4.554	1.028	0.134	TT
BLUP	MM50119	10	32584066	-0.673	6.019	1.337	0.282	TT
BLUP	MM53329	11	5245829	0.592	5.683	1.341	0.396	GG
BLUP	MM53925	11	9337224	0.781	6.664	2.096	0.326	AA
BLUP	MM60323	12	33108268	0.454	3.366	0.784	0.406	TT
BLUP	MM62878	13	11644260	-0.679	6.445	1.604	0.306	TT
BLUP	MM70818	14	33988495	0.707	3.602	1.256	0.158	GG
BLUP	MM70868	14	35215478	0.670	4.108	0.787	0.156	AA
BLUP	MM73028	15	3293035	0.599	6.260	1.405	0.480	CC
BLUP	MM76895	15	36572861	-3.159	11.655	3.069	0.018	GG
BLUP	MM87418	17	38233161	0.636	6.335	1.586	0.466	TT

r^2%: The proportion of phenotypic variance explained by each QTN; E1: Nanjing (2014); E2: Nanjing (2015); E3: Wuhan (2014); E4: Wuhan (2015).

Table S7: Significant QTNs for 100-seed weight detected in four environments and BLUP model by using MLM

Environment	SNP	Chr.	Position (bp)	P-value
E1	MM89014/qHSW-18-2	18	6705051	7.43E-07
E2	MM54178	11	11136355	4.26E-07
E3	MM51564/qcHSW-10-4	10	44349893	9.71E-08
E3	MM51565/ qcHSW-10-4	10	44349907	9.71E-08
E3	MM54178	11	11136355	3.97E-07
E4	MM33842	7	14760421	1.07E-06
E4	MM33843	7	14760422	1.07E-06
E4	MM67386	14	13165	1.04E-06
E4	MM75314	15	18149008	7.77E-07
E4	MM89014/SW50	18	6705051	1.61E-07
BLUP	MM54178	11	11136355	2.51E-07

E1: Nanjing (2014); E2: Nanjing (2015); E3: Wuhan (2014); E4: Wuhan (2015).

Table S8. Stable QTNs of soybean 100-seed weight identified in multiple environments and/or by multiple methods.

QTN or its cluster[a]	Position (bp)	Effect	LOD score	r2 (%)[b]	MAF	Method[c]	Environment[d]	SW increasing allele	Average 100-seed weight				SSR marker[f]
									SW increasing allele	SW decreasing allele	All the population	Significance[e]	
qcHSW-1-1	Gm01_6389301-6594737	1.36~4.07	4.71~6.30	2.93~4.94	0.09	1, 2, 3, 5, 6	E1~E3, BLUP	A	18.34~20.29	12.05~13.95	17.98~19.86	**	BARCSOYSSR_01_0337
qHSW-2-1	Gm02_11278769	0.52~1.97	4.14~5.86	1.01~3.10	0.38	2, 3, 4, 5	E4, BLUP	C	21.52	17.9	19.22	**	BARCSOYSSR_02_0586
qHSW-2-2	Gm02_43506875	0.83~2.77	3.14~5.66	1.06~2.53	0.13	1, 2, 3, 4, 5, 6	E1, E3, E4, BLUP	C	23.23~24.20	17.91~19.56	18.39~19.86	**	BARCSOYSSR_02_1373
qHSW-3-1	Gm03_17127049	0.76~1.49	3.86~6.09	0.61~1.67	0.1	2, 4	E1, E2, BLUP	G	22.50~24.89	17.45~17.68	17.98~18.39	**	BARCSOYSSR_03_0519
qHSW-3-2	Gm03_29644613	0.54~0.82	3.67~6.65	1.49~2.44	0.33	2, 5, 6	E1, E2, BLUP	T	19.12~19.48	16.05~16.51	17.98~18.39	**	BARCSOYSSR_03_0762
qHSW-3-3	Gm03_37435877	-2.55~-1.35	3.18~4.98	0.67~3.24	0.02	2, 5	E1, E4, BLUP	C	26.24~27.94	18.20~19.01	18.39~19.22	**	BARCSOYSSR_03_1155
qHSW-4-1	Gm04_6703334	-3.33~-0.48	3.01~8.64	1.05~5.34	0.2	1, 2, 3, 4, 5, 6	E1~E4, BLUP	C	21.37~24.09	17.20~18.93	17.98~19.22	**	BARC-025825-05102
qHSW-4-2	Gm04_37026887	0.59~1.71	3.34~9.93	1.11~4.20	0.26	1, 2, 3	E1~ E3, BLUP	A	19.29~21.24	14.54~16.33	17.98~19.86	**	BARCSOYSSR_04_0940
qHSW-4-3	Gm04_39207103	-1.19~-0.75	4.09~6.23	1.22~4.75	0.15	1, 2, 4, 5	E1, E2, E4	C	21.50~22.39	17.36~18.61	17.98~19.22	**	BARCSOYSSR_04_1006
qHSW-5-1	Gm05_38490635	0.62~1.24	3.29~8.13	1.24~2.94	0.47	2, 3	E1, E3, BLUP	A	19.64~20.73	17.00~18.85	18.39~19.86	*	BARCSOYSSR_05_1241
qHSW-5-2	Gm05_40410433	0.68~1.20	5.24~6.88	1.35~3.4	0.22	6	E1, E4, BLUP	A	19.44~20.16	14.29~15.53	18.39~19.22	**	BARC-007964-00162
qHSW-6-1	Gm06_5910218	0.71~2.71	3.50~11.68	1.61~6.94	0.4	1, 2, 3, 4, 5, 6	E1~ E3, BLUP	G	19.38~~21.49	15.71~17.24	17.39~19.86	**	BARC-045145-08894
qHSW-6-2	Gm06_8258824	1.06~1.52	3.44~5.49	1.50~3.04	0.06	1, 2, 4, 6	E4, BLUP	C	19.53	13.83	19.22	**	BARCSOYSSR_06_0451
qcHSW-6-3	Gm06_13385023-13385940	0.813~3.44	3.13~6.90	0.97~3.59	0.13	2, 3, 4, 5, 6	E1~ E3, BLUP	T	18.53~20.49	13.87~15.87	18.39~19.86	**	BARCSOYSSR_06_0614
qHSW-6-4	Gm06_14086552	0.45~2.37	3.03~9.43	0.63~5.13	0.38	1, 2, 3, 4, 5, 6	E1, E2, BLUP	C	19.16~19.99	15.81~16.07	17.98~18.39	**	BARCSOYSSR_06_0759
qHSW-7-1	Gm07_2535953	0.60~1.12	3.03~10.45	0.92~3.20	0.32	1, 2, 4, 6	E1, BLUP	C	19.95	15.22	18.39	**	BARCSOYSSR_07_0139
qHSW-7-2	Gm07_3954121	0.49~1.36	3.56~5.62	0.86~1.94	0.36	3, 5, 6	E2, BLUP	A	19.37	17.18	17.98	*	BARCSOYSSR_07_0206
qcHSW-7-3	Gm07_6439407-6463021	0.94~2.25	4.56~18.08	2.42~5.91	0.41	1, 2, 3, 4, 5, 6	E1~ E4, BLUP	T	19.74~21.58	16.79~18.74	17.98~18.39	**	BARCSOYSSR_07_0334
qHSW-7-4	Gm07_26301724	-3.48~-0.74	3.95~5.39	1.85~3.49	0.11	3, 5, 6	E4, BLUP	A	24.26	18.67	19.22	**	BARCSOYSSR_07_0989
qHSW-8-1	Gm08_10314889	0.46~0.76	3.17~4.33	0.68~1.79	0.28	1, 5, 6	E1, E2, E4, BLUP	C	19.97~21.47	17.27~18.40	17.98~19.22	**	BARC-038631-07266
qcHSW-10-1	Gm10_2563422-2566365	-3.22~-1.21	12.7~13.80	4.91~6.81	0.3	1, 2, 3, 4, 5, 6	E3, BLUP	C	20.73	18.04	19.86	**	BARCSOYSSR_10_0158
qcHSW-10-2	Gm10_32393792-32584066	-0.92~-0.62	4.75~6.01	1.26~2.51	0.28	1, 2, 5, 6	E4, BLUP	C	19.67	18.03	19.22	*	BARCSOYSSR_10_0902
qHSW-10-3	Gm10_42750933	0.98~1.50	3.96~10.75	2.54~5.89	0.23	2, 5, 6	E3	A	21.17	15.53	19.86	**	BARCSOYSSR_10_1330
qcHSW-10-4	Gm10_44349893-44469282	0.66~2.34	3.26~10.73	1.37~5.90	0.27	1, 2, 3, 4, 5, 6	E1~ E4, BLUP	C	20.08~21.88	17.23~19.28	17.98~19.22	**	BARCSOYSSR_10_1419

qHSW-11-1	Gm11_5245829	0.50~0.77	3.38~7.34	1.20~2.27	0.39	5, 6	E1, E2, BLUP	G	19.67~20.24	15.62~15.76	17.98~18.39	**	BARC-018099-02516
qHSW-11-2	Gm11_9337224	0.55~2.95	3.09~11.03	1.04~5.50	0.32	1, 2, 3, 4, 5, 6	E1, E2, BLUP	A	20.10~21.16	16.95~17.06	17.98~18.39	**	BARCSOYSSR_11_0511
qHSW-11-3	Gm11_11100801	-1.39~-0.75	3.19~5.25	1.34~2.88	0.12	5	E1, E2, E4, BLUP	A	22.35~24.23	17.38~18.54	17.98~19.22	**	BARCSOYSSR_11_0615
qHSW-11-4	Gm11_27803417	-1.36~-0.57	3.68~5.66	1.53~4.06	0.13	1, 2, 5	E1, E2	T	22.30~24.05	17.53~17.83	17.98~18.39	**	BARCSOYSSR_11_1051
qHSW-14-1	Gm14_10032197	0.79~1.52	3.77~15.34	1.36~6.90	0.25	4	E1, E2, BLUP	C	19.13~19.75	14.32~14.54	17.98~18.39	**	BARC-052759-11611
qHSW-14-2	Gm14_35215478	0.66~2.36	3.51~6.16	0.78~2.12	0.15	3, 4, 5, 6	E3, BLUP	A	24.66	18.99	19.86	**	BARCSOYSSR_14_1066
qHSW-15-1	Gm15_3293035	0.59~1.07	3.68~9.03	1.39~3.70	0.47	1, 2, 4, 5, 6,	E3, BLUP	C	20.96	18.68	19.86	*	BARCSOYSSR_15_0146
qHSW-16-1	Gm16_31087981	0.64~0.94	3.58~4.95	1.09~2.35	0.41	1, 2, 4	E1	A	19.69	17.59	18.39	**	BARCSOYSSR_16_1039
qHSW-16-2	Gm16_35442241	1.06~1.91	4.64~6.19	0.94~3.01	0.05	1, 2, 6	E2	G	23.36	17.69	17.98	**	BARCSOYSSR_16_1217
qHSW-17-1	Gm17_8760885	0.66~1.80	3.61~4.39	1.42~2.02	0.25	3, 4, 5	E1	G	19.68	14.86	18.39	**	BARCSOYSSR_17_0491
qHSW-17-2	Gm17_12908030	0.73~1.22	4.14~7.01	1.15~3.20	0.28	1, 2, 6	E1	T	20.56	17.72	18.39	**	BARCSOYSSR_17_0742
qHSW-17-3	Gm17_13325606	0.72~1.09	3.71~8.58	1.53~4.98	0.24	1, 2, 5	E4, BLUP	T	22.64	18.12	19.22	**	BARCSOYSSR_17_0762
qHSW-17-4	Gm17_16770188	-2.29~-0.83	4.81~13.31	2.59~6.13	0.37	1, 2, 3, 4, 5, 6	E3	A	20.87	18.24	19.86	**	BARCSOYSSR_17_0899
qHSW-17-5	Gm17_38229512	1.11~3.07	9.77~10.71	4.74~7.26	0.46	1, 3, 5	E3	A	21.23	18.25	19.86	**	BARCSOYSSR_17_1433
qHSW-17-6	Gm17_38985524	-3.38~-1.16	4.36~9.49	1.79~4.60	0.1	1, 2, 3, 4, 5, 6	E3, E4, BLUP	A	23.35~23.90	18.76~18.77	19.22~19.86	**	BARCSOYSSR_17_1474
qHSW-18-1	Gm18_5591131	1.17~1.51	6.16~12.84	2.69~4.36	0.18	1, 2, 5, 6	E3	C	20.48	17.22	19.86	**	BARCSOYSSR_18_0313
qHSW-18-2	Gm18_6705051	-3.98~-2.74	4.77~8.39	3.02~4.21	0.02	1, 4, 6	E1, E4	G	36.55~39.05	18.15~18.97	17.98~19.22	**	BARCSOYSSR_18_0370
qHSW-20-1	Gm20_30017454	0.52~1.10	3.51~8.38	0.95~4.37	0.32	1, 2, 4, 5	E2, BLUP	T	20.67	17.65	17.98	**	BARCSOYSSR_20_0654
qHSW-20-2	Gm20_45498156	0.76~1.82	3.26~10.33	1.93~5.09	0.37	1, 2, 3, 5	E1, E2, BLUP	G	19.20~20.07	16.33~16.63	17.98~18.39	**	BARC-047899-10425

[a] Stable QTNs (*qHSW*) and QTN clusters (*qcHSW*) which was identified in at least 3 environments/BLUP and/or by three ML-GWAS methods.

[b] r^2 (%) is a proportion of total phenotypic variation explained by each QTN.

[c] Methods mrMLM, FASTmrMLM, FASTmrEMMA, pLARmEB, pKWmEB, and ISIS EM-BLASSO were indicated 1-6, respectively.

[d] Environments E1, E2, E3, and E4 denote the population planted in Nanjing (2014), Nanjing (2015), Wuhan (2014), and Wuhan (2015), respectively.

[e] Significance at the 0.05 (*) and 0.01 (**) levels, using t-test, between SW increasing and SW decreasing alleles for each QTN.

[f] SSR markers located near the stable QTNs and these SSR markers were from the BARCSOYSSR_1.0 database of 33,065 SSRs [66]

Table S9: Distribution of SW increasing alleles in stable QTNs among 250 soybean accessions

QTN (or cluster)	Marker No.	Position (bp)	Elite allele	Percentage (%)[a]
qcHSW-1-1	MM936	Gm01_6389301-6594737	C	64.4
qHSW-2-1	MM7017	Gm02_11278769	C	36.4
qHSW-2-2	MM9632	Gm02_43506875	C	10.8
qHSW-3-1	MM12414	Gm03_17127049	G	7.20
qHSW-3-2	MM13399	Gm03_29644613	T	63.6
qHSW-3-3	MM14469	Gm03_37435877	C	2.40
qHSW-4-1	MM16825	Gm04_6703334	C	18.00
qHSW-4-2	MM19336	Gm04_37026887	A	72.00
qHSW-4-3	MM19598	Gm04_39207103	C	13.20
qHSW-5-1	MM25031	Gm05_38490635	A	52.40
qHSW-5-2	MM25289	Gm05_40410433	A	75.60
qHSW-6-1	MM26278	Gm06_5910218	G	58.00
qHSW-6-2	MM26594	Gm06_8258824	C	90.00
qcHSW-6-3	MM27416	Gm06_13385023-13385940	T	83.20
qHSW-6-4	MM27535	Gm06_14086552	C	61.20
qHSW-7-1	MM31764	Gm07_2535953	C	66.40
qHSW-7-2	MM31998	Gm07_3954121	A	36.00
qcHSW-7-3	MM32461	Gm07_6439407-6463021	T	40.80
qHSW-7-4	MM34903	Gm07_26301724	A	8.80
qHSW-8-1	MM38232	Gm08_10314889	C	27.20
qcHSW-10-1	MM47907	Gm10_2563422-2566365	C	67.60
qcHSW-10-2	MM50119	Gm10_32393792-32584066	C	68.80
qHSW-10-3	MM51364	Gm10_42750933	A	71.60
qcHSW-10-4	MM51564	Gm10_44349893-44469282	A	5.20
qHSW-11-1	MM53329	Gm11_5245829	G	57.60
qHSW-11-2	MM53925	Gm11_9337224	A	32.40
qHSW-11-3	MM54162	Gm11_11100801	A	12.00
qHSW-11-4	MM55590	Gm11_27803417	T	9.60
qHSW-14-1	MM68891	Gm14_10032197	C	74.40
qHSW-14-2	MM70868	Gm14_35215478	A	12.80
qHSW-15-1	MM73028	Gm15_3293035	C	47.20
qHSW-16-1	MM82685	Gm16_31087981	A	37.60
qHSW-16-2	MM83326	Gm16_35442241	G	5.60
qHSW-17-1	MM84793	Gm17_8760885	G	74.00
qHSW-17-2	MM85374	Gm17_12908030	T	25.60
qHSW-17-3	MM85419	Gm17_13325606	T	24.40
qHSW-17-4	MM85898	Gm17_16770188	A	62.00
qHSW-17-5	MM87416	Gm17_38229512	A	51.60
qHSW-17-6	MM87550	Gm17_38985524	A	10.00
qHSW-18-1	MM88823	Gm18_5591131	C	81.20
qHSW-18-2	MM89014	Gm18_6705051	G	1.20
qHSW-20-1	MM103406	Gm20_30017454	T	65.20
qHSW-20-2	MM105856	Gm20_45498156	G	57.20

[a] Percentage (%) was calculated as (total number of accessions containing SW increasing allele/total number of soybean accessions) × 100%.

Table S10. Predicted candidate genes for seed weight near the stable QTNs in soybean

Genome-wide association study		Soybean genes		Comparative genomic study			KEGG pathway
QTN (QTN cluster)	Position (bp)	Candidate gene	Position (bp)	Gene name	Arabidopsis gene	Functional annotation	
qHSW-2-1	Gm02_11278769	Glyma02g13120	Gm02:11340180-11345521	AFP2	AT1G13740	ABI five binding protein 2	
qHSW-3-3	Gm03_37435877	Glyma03g29431	Gm03:37412313-37417095	ADPG1	AT3G57510	Pectin lyase-like superfamily protein	Metabolic pathways, Pentose and glucuronate interconversions
qHSW-4-1	Gm04_6703334	Glyma04g08510	Gm04:6667096-6673224	ATSPP	AT2G03120	sigl peptide peptidase	
		Glyma04g08540	Gm04:6692425-6696307	LA1	AT5G07350	homologous La protein 1	
qHSW-4-3	Gm04_39207103	Glyma04g33610	Gm04:39240661-39242267	GASA4	AT5G15230	GAST1 protein homolog 4	
qHSW-5-1	Gm05_38490635	Glyma05g34030	Gm05:38495325-38497529	GmMFT		PEBP (phosphatidylethanolamine binding protein) family protein	
		Glyma05g34120	Gm05:38540979-38549756			Translation elongation factor EF1A	mRNA surveillance pathway
qHSW-6-1	Gm06_5910218	Glyma06g07940	Gm06:5854622-5855636	FLA8	AT2G45470	FASCICLIN-like arabinogalactan protein 8	
		Glyma06g08120	Gm06:5977269-5979353	WRKY11	AT4G31550.1	WRKY DNA-binding protein 11	
qHSW-6-2	Gm06_8258824	Glyma06g10830	Gm06:8199290-8204935	GmCRY1		cryptochrome 1	Circadian rhythm - plant
qcHSW-6-3	Gm06_13385023-13385940	Glyma06g16920	Gm06:13300048-13304817	GmCPK11		calcium-dependent protein kinase 2	Plant-pathogen interaction
		Glyma06g17020	Gm06:13364743-13371838	ERD1	AT5G51070	Clp ATPase	
		Glyma06g17050	Gm06:13404991-13413002	Tudor1	AT5G07350.1	TUDOR-SN protein 1	
qHSW-7-1	Gm07_2535953	Glyma07g03810	Gm07:2624510-2626840	GA3OX1	AT1G15550	gibberellin 3-oxidase 1	Diterpenoid biosynthesis, Biosynthesis of secondary metabolites
qHSW-7-2	Gm07_3954121	Glyma07g05250	Gm07:3902288-3905718	ARP1	AT3G54770	RNA-binding (RRM/RBD/RNP motifs) family protein	
		Glyma07g05260	Gm07:3911299-3912261	RTFL9	AT1G53708	ROTUNDIFOLIA like 9	
		Glyma07g05280	Gm07:3928005-3931812	GmPSY1R	AT1G72300	Leucine-rich receptor-like protein kinase family protein	
qcHSW-7-3	Gm07_6439407-6463021	Glyma07g07740	Gm07:6425997-6431837	EGL3	AT1G63650	basic helix-loop-helix (bHLH) DNA-binding superfamily protein	
		Glyma07g07850	Gm07:6497229-6504722	BSK3	AT4G00710	BR-signaling kinase 3	Plant hormone signal transduction
qcHSW-10-1	Gm10_2563422-2566365	Glyma10g03440	Gm10:2482369-2489108	MCCA	AT1G03090	methylcrotonyl-CoA carboxylase alpha chain	Metabolic pathways
		Glyma10g03470	Gm10:2531385-2537709	ATNEK4	AT3G63280	NIMA-related kinase 4	
qHSW-10-3	Gm10_42750933	Glyma10g34550	Gm10:42721979-42729453	CSLA9	AT5G03760	Nucleotide-diphospho-sugar transferases superfamily protein	
qcHSW-10-4	Gm10_44349893-	Glyma10g36070	Gm10:44258213-44261525	RPL21	AT1G35680	Ribosomal protein L21	Ribosome

	44469282	Glyma10g36200	Gm10:44376223-44379856	SUT1	AT1G22710	sucrose-proton symporter 2	
qHSW-11-1	Gm11_5245829	Glyma11g07550	Gm11:5273582-5277791	AUL1	AT1G75310	auxin-like 1 protein	
		Glyma11g07523	Gm11:5290144-5296775	RFC3	AT5G27740	ATPase family associated with various cellular activities (AAA)	DNA replication, Mismatch repair
qHSW-11-2	Gm11_9337224	Glyma11g12980	Gm11:9275328-9276819	GLB1	AT2G16060	hemoglobin 1	
		Glyma11g13200	Gm11:9395262-9403781	AP2	AT3G06160	AP2/B3-like transcriptional factor family protein	
		Glyma11g13210	Gm11:9405492-9409331	VRN1	AT3G18990	AP2/B3-like transcriptional factor family protein	
		Glyma11g13220	Gm11:9411946-9415396	AP2	AT3G06220	AP2/B3-like transcriptional factor family protein	
qHSW-11-3	Gm11_11100801	Glyma11g15490	Gm11:11128390-11130901	HERK1	AT3G46290	hercules receptor kise 1	
qHSW-15-1	Gm15_3293035	Glyma15g04790	Gm15:3363020-3366590	HERK1	AT3G46290	hercules receptor kise 1	
qHSW-17-2	Gm17_12908030	Glyma17g16210	Gm17:12901909-12904333	EXLB1	AT4G17030	expansin-like B1	
qHSW-17-3	Gm17_13325606	Glyma17g16620	Gm17:13354202-13355951	LEA4-1	AT1G32560	Late embryogenesis abundant protein	
qHSW-20-1	Gm20_30017454	Glyma20g21082	Gm20:30059997-30061296	AGL6	AT2G45650	AGAMOUS-like 6	
qHSW-20-2	Gm20_45498156	Glyma20g37550	Gm20:45403202-45405165		AT1G72210	basic helix-loop-helix (bHLH) D-binding superfamily protein	

qHSW and *qcHSW* denotes stable QTNs and QTN cluster, respectively.
